# METTL14 Promotes Chicken Preadipocyte Differentiation by Modulating mRNA Stability via a Dual-Reader m^6^A Mechanism

**DOI:** 10.3390/cells15191761

**Published:** 2026-09-27

**Authors:** Saibo Zhang, Qi Zhang, Yawen Yang, Chang Liu, Fei Teng, Shouzhi Wang, Hui Li, Bohan Cheng

**Affiliations:** 1 Key Laboratory of Chicken Genetics and Breeding, Ministry of Agriculture and Rural Affairs, Harbin 150030, China; s220501012@neau.edu.cn (S.Z.); zhangqi2022@byau.edu.cn (Q.Z.); s230501026@neau.edu.cn (Y.Y.); 1120200506@mail.nankai.edu.cn (C.L.); s240501013@neau.edu.cn (F.T.); shouzhiwang@neau.edu.cn (S.W.); 2 Key Laboratory of Animal Genetics, Breeding and Reproduction, Education Department of Heilongjiang Province, Harbin 150030, China; 3 College of Animal Science and Technology, Northeast Agricultural University, Harbin 150030, China; 4 Key Laboratory of Exploration and Innovative Utilization of White Goose Germplasm Resources in the Cold Region of Heilongjiang Province, #2 Xinyang Road, New Development District, Daqing 163319, China; 5 College of Animal Science and Technology, Heilongjiang Bayi Agricultural University, #2 Xinyang Road, New Development District, Daqing 163319, China

**Keywords:** chicken, adipogenesis, METTL14, m^6^A, mRNA stability, MeRIP-seq

## Abstract

**Highlights:**

**What are the main findings?**

- METTL14 promotes chicken preadipocyte differentiation by modulating mRNA stability of *BMI1*, *TMED3*, and *MFAP2* via a dual-reader (IGF2BP1/2 and YTHDF2) m^6^A mechanism.

**What is the implication of the main finding?**

- Our findings not only reveal the inter-species conservation of METTL14 function during preadipocyte differentiation; they also establish a mechanistic framework for the understanding of how an m^6^A methyltransferase achieves target-specific regulation through reader diversification in adipogenesis.

**Abstract:**

Preadipocyte differentiation is an essential process in adipogenesis. METTL14, an essential constituent of the *N*^6^-methyladenosine (m^6^A) methyltransferase complex, plays an important role in mammalian preadipocyte differentiation. However, its role in avian adipogenesis remains unclear. More importantly, the m^6^A mechanism by which METTL14 regulates preadipocyte differentiation has not been elucidated. Here, we found that the level of METTL14 expression was substantially elevated within the adipose tissue of the abdominal region of fat chickens relative to lean chickens. Knockdown of METTL14 inhibited preadipocyte differentiation, whereas overexpression of METTL14 promoted this process. Through the integration of MeRIP-seq and RNA-seq, we confirmed that *chBMI1*, *chTMED3*, and *chMFAP2* were functionally essential targets of METTL14-dependent m^6^A modification in adipogenesis. METTL14-mediated *chBMI1* mRNA stabilization and the degradation of *chTMED3* and *chMFAP2* mRNAs depend on IGF2BP1/2 and YTHDF2, respectively. Overall, our findings establish that METTL14 promotes chicken preadipocyte differentiation by modulating the stability of target mRNAs through a dual-reader m^6^A mechanism.

## 1. Introduction

Adipose tissue (AT) has the potential to expand in physiological conditions. AT expansion due to excessive calorie intake is a biologically appropriate reaction; however, obesity is associated with higher risks of developing diabetes and cardiovascular diseases [1]. Adipogenesis serves as the cellular base for the growth and development of ATs [2]. A complex network composed of multiple transcription factors (TFs), including C/EBPs, PPARγ, GATA2/3, and Kruppel-like factors (KLFs), regulates the differentiation of preadipocytes (preACs), a crucial phase of adipogenesis [3]. Further, the preAC differentiation is influenced by epigenetic changes, such as DNA methylation, chromatin remodeling, and alterations in histones [4]. RNA *N*^6^-methyladenosine (m^6^A) modifications are also involved in modulating preAC differentiation [5]. m^6^A is the predominant chemical modification found in mRNA, primarily located within the consensus binding motif of G [G > A] m^6^A C [U > A > C] [6]. It is dynamically regulated by the m^6^A methyltransferase complex (MTC), which consists of Wilms tumor protein 1-associated regulatory protein (WTAP) and methyltransferase-like proteins 14 and 3 (METTL14 and METTL3) [7,8], and is erased by m^6^A demethylases, AlkB homolog 5 (ALKBH5), and fat mass and obesity-associated protein [9,10]. Various RNA processing events, including mRNA structure [11], stability [12], splicing [13], transport [14], translation [15], and primary microRNA processing [16], are regulated by different reader proteins that recognize m^6^A-methylated transcripts.

METTL14 is a subunit of the MTC that cannot transfer methyl groups directly. However, it is crucial for recognizing RNA substrates and providing structural support to the catalytic cavity in METTL3 [17]. Arginine 298 in the human METTL14 plays a critical role in precisely recognizing RNA substrates by the METTL3/14 complex. The sequence-recognition specificity of the METTL3/14 complex for RNA substrate is modified by the mutation of R298 to proline (R298P), which also significantly reduces the complex’s m^6^A methylation activity [17]. Furthermore, the precise recognition of RNA targets is also dependent on the methylation of an arginine at the METTL14 C-terminus [18]. METTL14 has been implicated in various processes, including stem cell self-renewal [19], proliferation and differentiation of myoblasts [20], spermatogenesis [21], cell senescence [22], inflammation [23], atherosclerosis [24], and cancer [25]. Earlier investigations have indicated that the METTL14 positively regulates the differentiation of preACs in mice and bovines [26,27].

Chickens are among the most common agricultural livestock and are widely used as research models [28]. Globally, broiler production is primarily characterized by the use of fast-growing poultry. However, this rapid growth has been linked to increased abdominal fat deposition, which has resulted in various adverse outcomes, including affecting health during the laying period [29], reduced feed capability [30], reduced reproductive competence [31], and compromised meat quality [32]. Thus, controlling the deposition of abdominal fat is a key objective in poultry breeding programs. Our previous study analyzed DNA sequencing from Northeast Agricultural University broiler lines selected for abdominal fat content (NEAUHLF). This analysis revealed a crucial candidate gene, *chMETTL14*, substantially influencing chicken abdominal fat content [33]. Further, our previous findings revealed variations in DNA methylation of *chMETTL14* in abdominal AT between the two broiler lines [34], suggesting the involvement of this gene in chicken abdominal AT development. It was shown that there were differences in lipogenic capacity between mammals and birds [35]. Therefore, although the roles of METTL14 in preAC differentiation have been reported in mice and bovines, it is still necessary to explore its function in chicken adipogenesis. More importantly, the m^6^A mechanism by which METTL14 exerts its pro-adipogenic effect has not been determined. In this study, we assessed the role of METTL14 in chicken preAC differentiation and explored the underlying m^6^A mechanism.

## 2. Materials and Methods

### 2.1. Animals

Fifty-six 19th-generation (G_19_) (fat line, *n* = 28; lean line, *n* = 28) and 42 24th-generation (G_24_) (lean line, *n* = 21; fat line, *n* = 21) NEAUHLF male chickens were utilized. These lines have undergone selective breeding since 1996, based on plasma VLDL content and abdominal fat percentage (AFP). AFP was evaluated using the formula: AFP (%) = AFW/BW7 × 100%, in which AFW represents the weight of abdominal fat, and BW7 indicates the seven-week-old body weight. The breeding protocol was discussed previously [36]. The chickens were kept under constant conditions with unrestricted feed and water. Between hatching and at the age of three weeks, they were given an initial diet with 210 g/kg crude protein (CP) and 3000 kcal/kg metabolizable energy (ME), followed by a grower diet with 3100 kcal/kg ME and 190 g/kg CP between the ages of four and seven weeks.

### 2.2. Tissue Samples

For tissue harvesting, 56 male chickens from G_19_ (4 per line per week, ages 1–7 weeks) and 42 male birds from G_24_ (3 per line per week, ages 1–7 weeks) were slaughtered after a 12 h fasting period. AFP was measured as shown in Figure S1. Abdominal ATs were excised at the end of each week and washed with 0.75% NaCl before freezing using liquid N_2_ following preservation at a temperature equal to −80 °C.

### 2.3. Cell Culture and Induction of Differentiation

The immortalized chicken preAC cell line ICP2 (Cat. 4201MAM-CCTCC00703, China Center for Type Culture Collection, CCTCC) was previously established by our lab [37]. Primary chicken preACs were isolated from abdominal adipose tissue as previously described [38]. Briefly, samples of abdominal adipose tissue were isolated following rapid decapitation, and tissues were harvested aseptically from seven 10-day-old broilers. The collected adipose tissues were treated with pre-warmed PBS to remove blood and debris, which were provided with streptomycin (100 μg/mL; Gibco, Grand Island, NY, USA) and penicillin (100 units/mL); dissected with specialized autoclaved surgical scissors; and subjected to digestion in 2 mg/mL collagenase type I (Gibco), with constant agitation for 65 min at 37 °C. The suspension was filtered through a 100 and 20 μm mesh (BD Falcon, Bedford, MA, USA) and subjected to centrifugation at 300× *g* for 10 min at room temperature to separate the stromal-vascular cells (primary chicken preACs) from undigested tissue debris and mature adipocytes. ICP2 cells or primary chicken preACs were maintained in DMEM/F12 (Gibco) supplemented with 10% fetal bovine serum (FBS; Biological Industries, Kibbutz Beit Haemek, Israel), at a temperature equal to 37 °C, with 5% CO_2_. When cultures reached approximately 90% confluence, they were either passaged or seeded at 6 × 10^4^ cells/mL for further experiments. Upon reaching an appropriate density, differentiation was induced using a fresh induction medium containing 160 μM oleate in DMEM/F12 supplemented with 10% FBS.

### 2.4. Construction of a Preadipocyte Cell Line Stably Overexpressing METTL14

The construction of lentiviral vectors carrying the METTL14 overexpression construct (LV-METTL14) and a corresponding negative control (LV-control) was conducted by Hanbio (Shanghai, China) and utilized to infect ICP2 cells (LV-METTL14 MOI: 200; LV-control MOI: 200), with selection with blasticidin (4 mg/mL) starting 72 h post-infection and being continued for 8 days to isolate resistant clones. Green fluorescent protein (GFP)-positive cells were identified by performing flow cytometry, followed by seeding of individual cells in 96-well plates to establish monoclonal populations. Stable overexpression of METTL14 was verified by Western blotting.

### 2.5. RNA Interference

All siRNAs targeting *chMETTL14*, B-cell-specific Moloney murine leukemia virus insertion site 1 (*chBMI1*), transmembrane emp24 protein transport domain containing 3 (*TMED3*), microfibrillar-associated protein 2 (*chMFAP2*), YTH *N*^6^-methyladenosine RNA-binding protein F2 (*chYTHDF2*), insulin-like growth factor 2 mRNA-binding protein 1 (*chIGF2BP1*), *chIGF2BP2*, *chIGF2BP3*, and negative controls were synthesized by GenePharma (Shanghai, China). These siRNAs are referred to as si-METTL14, si-BMI1, si-TMED3, si-MFAP2, si-YTHDF2, si-IGF2BP3, si-IGF2BP2, si-IGF2BP1, and si-NC, respectively. The siRNA sequences are shown in Table S1.

Primary chicken preACs were grown in 12-well plates. After 60% cell confluency, transfection of effective si-METTL14 or si-NC into primary chicken preACs was performed by utilizing Lipofectamine 3000 reagent (Invitrogen, Carlsbad, CA, USA) as per the established protocol. ICP2 cells were inoculated in 12-well plates. When cells were 60% confluent, transfection of effective si-METTL14, si-BMI1, si-TMED3, si-MFAP2, or si-NC into ICP2 cells was performed by utilizing Lipofectamine 3000 reagent (Invitrogen) as per the established protocol. LV-METTL14 and LV-control cells were plated in 12-well plates for the rescue experiment. Similarly, after 60% cell confluency, effective si-BMI1, si-IGF2BP1, si-IGF2BP2, si-YTHDF2, or si-NC was transfected into LV-METTL14 or LV-control cells using Lipofectamine 3000 reagent (Invitrogen) as per the established protocol. Then the cells were induced for differentiation, and the interference efficiency of the effective siRNAs was further verified during the differentiation process.

### 2.6. Oil Red O (ORO) Staining and Quantification

Lipid droplet formation was visualized using ORO (Sigma-Aldrich, St. Louis, MO, USA) staining. Following three washes in PBS, fixation for 30 min in 4% paraformaldehyde, and three further PBS washes, cells underwent staining with ORO at ambient temperature for 15 min, followed by rinsing in PBS to remove excess stain. To remove background staining, cells were briefly treated with 60% isopropanol for 10–20 s, followed by washing with distilled water. Stained lipid droplets were observed and imaged under inverted fluorescence microscopy (Leica Microsystems, Wetzlar, Germany).

For quantification, an ORO extraction assay was accomplished as defined earlier [39]. Elution of the dye retained in the stained lipid droplets was achieved with 100% isopropanol, and absorbance at 510 nm was measured. Prior to the staining procedure, the number of cells in each group was estimated using CellTiter 96^®^ AQueous One Solution assays (Promega, Madison, WI, USA), according to 490 nm absorbance. Lipid accumulation levels were normalized to cell numbers and expressed as the OD_510_/OD_490_ ratio.

### 2.7. RT-qPCR

The extractions of total RNA from the cells and tissues were accomplished by using TRIzol (Invitrogen). After synthesizing cDNA using a PrimeScript RT reagent kit with gDNA Eraser (Takara, Beijing, China), qPCR was conducted by employing the FastStart Universal SYBR Green Master kit (Roche, Penzberg, Germany), using the conditions of 95 °C for a duration equal to 10 min, 40 cycles at a temperature equal to 95 °C for a duration equaling 1 min, and 60 °C for a duration equal to 1 min. Threshold cycle (Ct) values were determined as the arithmetic mean of technical replicates, and relative gene expression was computed using the 2^−ΔCt^ method, with TATA-box-binding protein (*TBP*) as the internal control [40]. Table S2 illustrates the primers used for RT-qPCR.

### 2.8. Western Blot (WB)

The isolation of total protein from the cells and tissues was performed by utilizing RIPA buffer (Beyotime, Shanghai, China). The total protein concentration of each sample was measured using a BCA protein assay kit (Invitrogen) according to the manufacturer’s instructions. After that, equal amounts of total protein were loaded and separated using a 12% gel SDS/PAGE and subsequently electroblotted to PVDF membranes (Millipore, Billerica, MA, USA). For the blocking step, the blots were immersed in PBST with 5% non-fat milk for 4 h at ambient temperature and then treated with METTL14 rabbit polyclonal antibody (1:1000; Abmart, Shanghai, China), HA-tag mouse monoclonal antibody (1:1000; Beyotime), Myc-tag mouse monoclonal antibody (1:1000; Beyotime), or β-actin mouse monoclonal antibody (1:1000; Beyotime) at a temperature equal to 4 °C for a duration equaling 12 h, followed by the respective HRP-labeled IgG (1:5000; Beyotime) for a duration equal to 1 h at room temperature. Ultimately, the bands of interest were observed and examined using a Tanon-5200 chemiluminescence analyzer from Tanon Science and Technology Co., Ltd. (Shanghai, China). Bands were assessed via densitometric analysis with ImageJ (NIH, Bethesda, MD, USA).

### 2.9. m^6^A Dot Blot Assay

Total RNA (200 ng), pretreated with DNase I (Sigma), was added dropwise onto a nylon membrane (Thermo Fisher, Waltham, MA, USA) and crosslinked under UV light (254 nm, 1200 J) for 1 min at ambient temperature. Membranes were blocked as previously discussed and treated overnight at a temperature equal to 4 °C with anti-m^6^A antibody (1:2000; Synaptic Systems, Göttingen, Germany). Blots were treated at ambient temperature for 1 h with the HRP-linked secondary antibody (1:5000) (Beyotime). Visualization was undertaken with an ECL reagent (HaiGene, Harbin, China) with a chemiluminescence system, with quantification using ImageJ 1.46r.

### 2.10. MeRIP-Seq and RNA-Seq

LC-Bio Technologies (Hangzhou, China) performed MeRIP-seq using ICP2 cells stably expressing METTL14 (LV-METTL14, *n* = 3) and their corresponding controls (LV-control, *n* = 3). Poly(A)-enriched RNA was fragmented and treated with an anti-m^6^A antibody (Synaptic Systems) within IP buffer (50 mM Tris-HCl, 750 mM NaCl, and 0.5% Igepal) at a temperature equal to 4 °C for a duration equaling 2 h. Immunoprecipitated RNA was subjected to reverse transcription to cDNA using SuperScript™ II Reverse Transcriptase (Invitrogen) before synthesis of U-labeled second-strand DNA with RNase H (NEB), *E. coli* DNA polymerase I (NEB), and dUTP solution (Thermo Fisher). Following end repair and A-tailing, DNA fragments were ligated to indexed adapters with T overhangs using AMPure XP beads for size selection. U-labeled second strands were removed with heat-labile UDG (NEB), and libraries were PCR-amplified (3 min at 95 °C; 8 cycles at a temperature equal to 98 °C for a duration equaling 15 s, 60 °C for a duration equal to 15 s, and 72 °C for a duration equaling 30 s; and, finally, 72 °C for a time of 5 min). The resulting cDNA libraries (average insert size 300 ± 50 bp) were sequenced as 2 × 150 bp paired-end reads on the Illumina NovaSeq 6000 platform (LC-Bio Technologies, China).

Adapter-contaminated, low-quality, and ambiguous reads were removed using fastp, followed by alignment of clean reads to the GRCg6a chicken reference genome using HISAT2. m^6^A peaks were called using exomePeak in R (FDR < 0.05), and the peaks were annotated to RefSeq (GRCg6a). Motif discovery was performed using MEME and HOMER, and differential m^6^A peaks were identified (FC > 1.2; *p* < 0.05) by comparing IP and input samples using exomePeak. Selected MeRIP-seq targets were verified by MeRIP-qPCR as previously described [41], and primer sequences are given in Table S3. Particularly, MeRIP-qPCR assay of *chBMI1*, *chTMED3*, or *chMFAP2* was conducted in LV-METTL14 cell line, LV-control cell line, and primary chicken preadipocytes transfected with pCMV-HA-METTL14 vector or pCMV-HA empty vector using MeRIP kit (Bersinbio, Guangzhou, China). pCMV-HA-METTL14 vector was constructed by Genewiz company (Genewiz, Suzhou, China).

Total RNA for RNA-seq was isolated from LV-METTL14 (*n* = 3) and LV-control (*n* = 3) cells using TRIzol. Construction and sequencing of the libraries were undertaken on an Illumina HiSeq 4000 system. After quality filtering and alignment with the GRCg6a reference genome, differentially expressed genes (DEGs) were evaluated using thresholds of FC > 1.2 and FDR < 0.05.

### 2.11. Luciferase Reporter Assays

The appropriate 3′UTR fragments of *chBMI1*, *chTMED3*, and *chMFAP2* containing the wild-type m^6^A and mutant (m^6^A was replaced by T) motifs were synthesized and inserted downstream of Renilla luciferase of the psi-Check2 vector (Genewiz). These constructs were transfected into LV-METTL14 or LV-control cells in a 24-well plate for 48 h, after which luciferase activity was assessed with a Dual-Luciferase Assay System (Promega).

### 2.12. RNA Stability Assay

For *chBMI1*, *chTMED3*, or *chMFAP2* mRNA stability analysis, LV-METTL14 and LV-control cells were grown in 6-well plates with 5 μg/mL actinomycin D for a duration equal to 0, 3, 6, or 12 h. Total RNA was then extracted from the cells for RT-qPCR analysis. Table S4 illustrates the primers used for RT-qPCR.

For mRNA stability analysis of the *Renilla* luciferase gene with wild-type and m^6^A site mutant 3′ UTR of *chBMI1*, *chTMED3*, or *chMFAP2*, the reporter constructs were transfected into LV-METTL14 or LV-control cells in a 12-well plate for 24 h. Then, the cells were treated with 5 μg/mL actinomycin D for 0, 3, 6, or 12 h. Total RNA was then extracted from the cells for luciferase reporter-related gene-specific RT-qPCR analysis. Table S2 illustrates the primers used for luciferase reporter-related gene-specific RT-qPCR.

### 2.13. Luciferase Reporter-Related Gene-Specific m^6^A-qPCR

To access whether m^6^A presents on the luciferase reporter constructs and further confirm whether METTL14 participates in regulating m^6^A modification of the constructs bearing *chBMI1*, *chTMED3*, and *chMFAP2* 3′UTR, we conducted luciferase reporter-related gene-specific m^6^A-qPCR. Primarily, luciferase reporter constructs, including BMI1-3′UTR-WT, BMI1-3′UTR-Mut, TMED3-3′UTR-WT, TMED3-3′UTR-Mut, MFAP2-3′UTR-WT, and MFAP2-3′UTR-Mut, were transfected into LV-METTL14 or LV-control cells in a 15 cm plate. Six hours following transfection, the cells were induced to differentiate. After 48 h of differentiation, isolation of the sample was completed, following the extraction of RNAs, m^6^A RNA immunoprecipitation assay was conducted and qPCR was performed to assess m^6^A levels with primers covering the joints between inserted *chBMI1*, *chTMED3* and *chMFAP2* 3′UTR and psi-Check2 vector. Table S4 illustrates the primers used for luciferase reporter-related gene-specific m^6^A-qPCR.

### 2.14. RNA Immunoprecipitation (RIP) Assay

RIP was performed using a RIP Kit (BersinBio) according to the manufacturer’s protocols. For RIP assays of endogenous mRNA, pCMV-Myc-IGF2BP1, pCMV-Myc-IGF2BP2, or pCMV-Myc-YTHDF2 construct was transfected into LV-METTL14 cells in a 15 cm plate; for RIP assays of exogenous mRNA, luciferase reporter constructs, including BMI1-3′UTR-WT, BMI1-3′UTR-Mut, TMED3-3′UTR-WT, TMED3-3′UTR-Mut, MFAP2-3′UTR-WT, and MFAP2-3′UTR-Mut, were transfected into LV-METTL14 cells in a 15 cm plate. At 6 h following transfection, the cells were induced to differentiate. After 48 h of differentiation, following collection, cells were lysed in lysis buffer, after which the lysates were incubated with magnetic beads coated with anti-Myc (Proteintech, Wuhan, China), anti-HA (Abcam, Cambridge, UK), or anti-IgG (Bes5101, BersinBio, Guangzhou, China) antibody. Afterward, the immunoprecipitated RNAs were isolated and purified. The coprecipitated RNAs were then detected using RT-qPCR. Table S5 illustrates the primers used for RIP-qPCR.

### 2.15. Statistical Analysis

Data were analyzed using JMP v11.0 (SAS Institute, Inc., Cary, NC, USA) and presented as mean ± standard deviation (SD). For data involving a single independent variable with two-group comparisons, independent sample *t*-test was used. For data involving two independent variables with two-group comparisons, two-way ANOVA, followed by Sidak’s post hoc test, was employed. For data involving a single independent variable with multiple-group comparisons, one-way ANOVA, followed by Tukey’s HSD post hoc test, was employed. The level of significance threshold was *p* < 0.05.

## 3. Results

### 3.1. METTL14 Is Highly Expressed in AT of Fat Chickens and Downregulated During preAC Differentiation

Firstly, the *chMETTL14* mRNA levels in abdominal AT from the fat and lean broilers (1 to 7 weeks) were examined. At 1, 3, 4, 5, and 6 weeks, *chMETTL14* mRNA levels were substantially higher in abdominal ATs of obese broilers relative to lean broilers (*p* < 0.05) (Figure 1A). Then, the METTL14 protein levels in abdominal AT from the fat and lean broilers (2, 3, 4, and 6 weeks) were examined. METTL14 protein levels at 2 and 4 weeks were markedly greater in ATs of fat relative to lean broilers (*p* < 0.05) (Figure 1B,C). Next, to determine whether METTL14 was implicated in chicken adipogenesis, METTL14 expression was analyzed during ICP2 cell differentiation. In ORO staining and RT-qPCR, both the concentrations of lipid droplets and *chPPARγ* and adipocyte fatty acid-binding protein (*chA-FABP*) increased as differentiation progressed, suggesting that the cells were undergoing a normal differentiation process (Figure 1D–G). RT-qPCR and WB experimental findings suggest that METTL14 expression was reduced during ICP2 cell differentiation (Figure 1H–J). The current results demonstrate that METTL14 may be critical to adipose deposition and preAC differentiation in chickens.

### 3.2. METTL14 Positively Regulates Chicken preAC Differentiation

To examine the functions of METTL14 in preAC differentiation in chicken, an effective and specific *chMETTL14* interference fragment (Figure S2) was transfected into the ICP2 cells. At 0, 24, 48, and 72 h of induced differentiation, the effect of METTL14 knockdown on preadipocyte differentiation was evaluated by lipid droplet deposition and the expression of adipogenic marker genes. METTL14 knockdown inhibited accumulation of lipid droplets at 48 and 72 h after induction of differentiation (*p* < 0.01; Figure 2A–E) and mRNA levels of *chC/EBPα* (24 and 48 h), *chPPARγ* (24 to 72 h), and *chA-FABP* (24 to 72 h) (*p* < 0.01 or *p* < 0.05) (Figure 2F). To avoid cell line-specific biases, we analyzed the effect of METTL14 knockdown on adipogenesis in primary chicken preadipocytes at 0, 24, 48, and 72 h of induced differentiation. Similar to ICP2 cells, METTL14 knockdown inhibited lipid droplet accumulation at 48 and 72 h (*p* < 0.05 or *p* < 0.01; Figure 3A–D) and the mRNA levels of *chPPARγ* (48 and 72 h) and *chA-FABP* (48 and 72 h) (*p* < 0.05 or *p* < 0.01) (Figure 3E). The current findings revealed that the knockdown of METTL14 inhibited chicken preAC differentiation.

The biological role of METTL14 in chicken preACs differentiation was also evaluated by lentivirally transducing HBLV-GFP-HA-METTL14 or HBLV-GFP-HA into ICP2 cells. Stable clones were selected using blasticidin (4 mg/mL) and flow cytometry. One METTL14-overexpressing (LV-METTL14) and one control (LV-control) stable preAC line were selected. Next, the WB analysis revealed the overexpression of METTL14 in the LV-METTL14 cell line. The results showed that the LV-METTL14 cell line exhibited approximately a two-fold amplification in METTL14 protein level compared with LV controls (*p* < 0.01) (Figure 4A,B). It was also investigated whether the global m^6^A level was altered by METTL14 overexpression, as it is the central component of the *N*^6^-methytransferase complex. The global m^6^A level in the LV-METTL14 cell line was considerably greater than that of the LV control cells, according to the m^6^A dot blot analysis (*p* < 0.01) (Figure 4C,D). The findings showed that the chicken preAC cell line that overexpressed METTL14 was effectively established.

Next, the effects of METTL14 overexpression on the preAC differentiation were analyzed. Overexpression of METTL14 significantly increased lipid deposition in the cells at 48 and 72 h after differentiation induction (*p* < 0.01) (Figure 4E,F), and the levels of *chC/EBPα* (72 h), *chPPARγ* (24 to 72 h), and *chA-FABP* (24 to 72 h) were considerably upregulated (*p* < 0.01 or *p* < 0.05; Figure 4G). Together, the presented observations illustrated that METTL14 positively controlled the chicken preACs differentiation.

### 3.3. chBMI1, chTMED3, and chMFAP2 Are Critical Targets of METTL14-Dependent m^6^A Modification

To examine the regulatory function of METTL14 in adipogenesis, RNA-seq was utilized to assess gene profiles in LV-METTL14 and LV-control cells 72 h after inducing differentiation. RNA-seq analysis revealed 1763 upregulated and 1177 downregulated genes in response to METTL14 overexpression (FC > 1.2; FDR < 0.05) (Figure 5A and Data S1). The m^6^A methylomes in LV-METTL14 and LV-control cells were then mapped via MeRIP-seq 72 h after inducing differentiation. In METTL14-overexpressing and control cells, 15,066 and 15,265 m^6^A peaks corresponding to 9525 and 9556 transcripts were identified, respectively (Data S2 and Data S3). The canonical GGACU sequence was significantly enriched in m^6^A sites (Figure 5B), and these peaks were predominantly located near the start and stop codons, as well as near the beginning of 3′ UTRs (Figure 5C). MeRIP-seq revealed that the abundance of m^6^A peaks in 270 genes increased (FC > 1.2; *p* < 0.05) (Data S4). Interestingly, the MeRIP-seq and RNA-seq analysis showed an overlap of 60 genes (Figure 5D). Of the above 60 genes, stearoyl-CoA desaturase (*SCD*) [42], neuronal PAS domain protein 3 (*NPAS3*) [43], natriuretic peptide receptor 2 (*NPR2*) [44], *BMI1* [45], *MFAP2* [46], and *TMED3* [47,48] have been reported to be involved in regulating adipogenesis or may be related to adipogenesis; therefore, they were chosen as potential targets of METTL14-dependent m^6^A modification for further investigation.

Next, RT-qPCR was utilized to confirm the RNA-seq results of the six candidate targets of METTL14. Except for *chSCD*, the expression of the other five genes aligned with the RNA-seq data (Figure 5E). Then, we conducted MeRIP-qPCR on the five genes to verify the MeRIP-seq data. The mRNA m^6^A methylation of the four genes, excluding *chNPR2*, aligns with the MeRIP-seq data (Figure 5F). These results confirmed both the MeRIP-seq and RNA-seq data. To further screen the key targets of METTL14 from the above four genes, the mRNA levels of *chMETTL14*, *chBMI1*, *chNPAS3*, *chTMED3*, and *chMFAP2* were examined in the AT of lean and fat broilers aged one to seven weeks, and a correlation analysis was conducted. *chBMI1* shows a remarkable positive correlation with *chMETTL14* expression (Figure 5G), while *chTMED3* and *chMFAP2* exhibit a significant inverse correlation with *chMETTL14* expression (Figure 5I,J) in chicken abdominal AT. No correlation was found between *chMETTL14* and *chNPAS3* levels (Figure 5H). These results suggest that three genes (*chBMI1*, *chTMED3*, and *chMFAP2*) may be important targets for METTL14. Furthermore, to avoid cell line-specific biases, we analyzed the effects of overexpression of METTL14 on the expression levels and m^6^A levels of *chBMI1*, *chTMED3*, and *chMFAP2* in primary chicken preadipocytes. Similar to the ICP2 cell line, METTL14 overexpression upregulated *chBMI1* but downregulated *chTMED3* and *chMFAP2* (Figure S3A); overexpression of METTL14 increased the m^6^A levels of *chBMI1*, *chTMED3*, and *chMFAP2* (Figure S3B). Collectively, *chBMI1*, *chTMED3*, and *chMFAP2* were identified as the critical METTL14 targets.

### 3.4. METTL14 Stabilizes chBMI1 mRNA and Reduces mRNA Stability of chTMED3 and chMFAP2 via the m^6^A-IGF2BPs/YTHDF2-Dependent Manner

Based on the MeRIP-seq data, the m^6^A peaks found within the *chBMI1*, *chTMED3*, and *chMFAP2* 3′ UTRs were markedly enhanced by the METTL14 overexpression (Figure S4 and Table S6). The bioinformatic analysis results of the m^6^A peaks depicted that there were three m^6^A sites in the *chBMI1* 3′ UTR, one m^6^A site in the *chTMED3* 3′ UTR, and two m^6^A sites in the *chMFAP2* 3′ UTR (Figure 6A). Luciferase reporter assays demonstrated that overexpression of METTL14 substantially increased luciferase activity of the reporter construct with WT *chBMI1* 3′ UTR with intact m^6^A sites, while the promotion was disrupted by mutations in the all three m^6^A sites (Mut1,2,3) or m^6^A site-1 (Mut1) (Figure 6B); METTL14 overexpression substantially lowered the activity of WT *chTMED3* 3′ UTR construct, whereas mutation in the m^6^A site abolished the inhibition (Figure 6B); overexpression of METTL14 substantially decreased the activity of WT *chMFAP2* 3′ UTR construct, while mutations in both m^6^A sites (Mut1,2) or m^6^A site-1 (Mut1) abrogated the inhibition (Figure 6B). These results indicate that METTL14-mediated gene regulation relies on the m^6^A modifications. Importantly, the methylation of the m^6^A site in the 3′ UTR of *chBMI1* (A2773), *chTMED3* (A1221), and *chMFAP2* (A850) participates in the METTL14-mediated regulation of the target genes, respectively. To demonstrate m^6^A placement by METTL14 on the exact site within the 3′UTR of *chBMI1*, *chTMED3*, or *chMFAP2*, we conducted METTL14 RIP-qPCR and gene-specific m^6^A-qPCR assay of the *Renilla* luciferase gene with the wild-type and m^6^A site mutant 3′ UTR of *chBMI1*, *chTMED3*, or *chMFAP2* in LV-control and LV-METTL14 cells. Data analysis of RIP-qPCR revealed that METTL14 could interact with the mRNA of the *Renilla* luciferase gene with the wild-type 3′ UTR of *chBMI1*, *chTMED3*, or *chMFAP2*, but it could not bind to the mRNA of the *Renilla* luciferase gene with the mutant 3′ UTR of *chBMI1* (A2773T), *chTMED3* (A1221T), or *chMFAP2* (A850T) (Figure 6C). The results of luciferase reporter-related gene-specific m^6^A-qPCR showed that METTL14 overexpression substantially increased the m^6^A level of the *Renilla* luciferase gene with wild-type 3′ UTR of *chBMI1*, *chTMED3* or *chMFAP2*, while the promotion was eliminated by mutation of the m^6^A site (BMI1 3′ UTR-A2773T, TMED3 3′ UTR-A1221T, and MFAP2 3′ UTR-A850T) (Figure 6D).

Next, the effect of METTL14 on the mRNA stability of *chBMI1*, *chTMED3*, and *chMFAP2* was investigated. The findings showed that METTL14 overexpression enhanced the stability of the *chBMI1* mRNA transcript, while *chTMED3* and *chMFAP2* mRNA transcripts were destabilized (Figure 6E). To rule out non-m^6^A-dependent effects of METTL14 on mRNA turnover, we conducted mRNA stability analysis of the *Renilla* luciferase gene with wild-type and m^6^A site mutant 3′ UTR of *chBMI1*, *chTMED3*, or *chMFAP2* in LV-control and LV-METTL14 cells. These findings demonstrated that METTL14 overexpression substantially increased the mRNA stability of *Renilla* luciferase gene with WT BMI1 3′ UTR, while the promotion was eliminated by mutation of the m^6^A site (BMI1 3′ UTR-A2773T); METTL14 overexpression substantially lowered the mRNA stability of *Renilla* luciferase gene with WT TMED3 or MFAP2 3′ UTR, whereas mutation of the m^6^A site (TMED3 3′ UTR-A1221T and MFAP2 3′ UTR-A850T) abolished the inhibition (Figure 6F,G). Therefore, the upregulation of *chBMI1* and downregulation of *chTMED3* and *chMFAP2* caused by METTL14 are at least partially attributed to the enhanced stability of *chBMI1* mRNA transcript and reduced stability of *chTMED3* and *chMFAP2* mRNA transcripts, respectively, resulting from the METTL14-dependent increase in m^6^A level within their mRNA transcripts.

Previous studies have shown that m^6^A reader proteins IGF2BPs (IGF2BP1, IGF2BP2, and IGF2BP3) and YTHDF2 could recognize a set of m^6^A-modified transcripts, increasing and decreasing the RNA stability, respectively [12,49]. Interestingly, the deletion of IGF2BP1 or IGF2BP2, but not IGF2BP3, substantially lowered the expression of *chBMI1* (Figure 7A and Figure S2), while suppression of YTHDF2 substantially raised *chTMED3* and *chMFAP2* expression (Figure 7B and Figure S2). Further, mRNA stability analysis showed that knockdown of IGF2BP1/2 decreased *chBMI1* mRNA stability, whereas knockdown of YTHDF2 increased the mRNA stability of *chTMED3* and *chMFAP2* (Figure 7C). For the demonstration of the physical attachment of IGF2BP1/2 to *chBMI1* transcript and the physical binding of YTHDF2 to the *chTMED3* and *chMFAP2* transcripts, we conducted RIP-qPCR assays in LV-METTL14 cells expressing the pCMV-Myc-IGF2BP1, pCMV-Myc-IGF2BP2, or pCMV-Myc-YTHDF2 constructs. The experimental findings of the RIP assay indicated that IGF2BP1/2 could bind to the *chBMI1* transcript, and YTHDF2 could bind to *chTMED3* and *chMFAP2* transcripts (Figure 7D). Further, the deletion of IGF2BP1 or IGF2BP2 reversed the upregulation of *chBMI1* triggered by METTL14 (Figure 7E). Similarly, the suppression of YTHDF2 weakened the downregulation of *chTMED3* and *chMFAP2* induced by METTL14 (Figure 7F). Collectively, METTL14 stabilizes *chBMI1* mRNA via an m^6^A-IGF2BP1/2-dependent manner, while it suppresses mRNA stability of *chTMED3* and *chMFAP2* via a pathway that depends on m^6^A-YTHDF2.

### 3.5. chBMI1, chTMED3, and chMFAP2 Are Functionally Essential Targets of METTL14-Induced Preadipocyte Differentiation

To further investigate the biological role of BMI1, TMED3, and MFAP2 in preAC differentiation, an effective and specific *chBMI1*, *chTMED3*, or *chMFAP2* interference fragment (Figure S2) was transfected into the ICP2 cells. The *chBMI1*-knockdown cells showed reduced lipid droplet accumulation (*p* < 0.05) (Figure 8A) and *chPPARγ* and *chA-FABP* mRNA levels (*p* < 0.01 or *p* < 0.05) (Figure 8B); meanwhile, knockdown of *chTMED3* or *chMFAP2* promoted accumulation of lipid droplets (*p* < 0.01 or *p* < 0.05) (Figure 8C,E) and mRNA levels of *chPPARγ* and *chA-FABP* (*p* < 0.05) (Figure 8D,F). The current outcomes showed that suppression of BMI1 can inhibit preAC differentiation, while knockdown of TMED3 or MFAP2 can enhance this process.

Subsequently, functional rescue experiments investigated whether BMI1, TMED3, and MFAP2 could mediate the regulative impact of METTL14 on preAC differentiation. It was found that the stimulating effect of METTL14 overexpression on lipid droplet accumulation and the mRNA level of *chPPARγ* and *chA-FABP* was attenuated by the suppression of *chBMI1* (*p* < 0.05 or *p* < 0.01) (Figure 9A,B). Overexpression of *chTMED3* or *chMFAP2* (Figure S5) restricted the stimulating effect of METTL14 overexpression on lipid droplet accumulation (*p* < 0.05 or *p* < 0.01) (Figure 9C,E) and the levels of *chPPARγ* and *chA-FABP* (*p* < 0.05) (Figure 9D,F). These results indicate that *chBMI1*, *chTMED3*, and *chMFAP2* are functionally important targets, at least in part, mediating the pro-adipogenic role of METTL14 during preAC differentiation.

## 4. Discussion

PreAC differentiation has been extensively investigated in terms of epigenetic regulation, particularly DNA and histone modifications, but little is known of RNA modifications [4]. Currently, over 100 distinct post-transcriptional RNA modifications are known, among which m^6^A accounts for more than 80%. m^6^A is critical in controlling pre-mRNA splicing, mRNA stability, microRNA processing, and translational efficiency [50]. METTL14 is a key component of the MTC. Although the roles of METTL14 in preAC differentiation have been reported in mice and bovines [26,27], METTL14’s function in birds is not clear. More importantly, the m^6^A mechanism by which METTL14 regulates preAC differentiation remained unidentified. In the current study, we determined that METTL14-dependent m^6^A modification promotes chicken preAC differentiation by increasing *chBMI1* mRNA stability and decreasing the mRNA stability of *chTMED3* and *chMFAP2*, which were modulated by IGF2BP1/2 and YTHDF2, respectively.

Currently, there is limited research on the pattern of expression of METTL14 in AT and adipogenesis. Heng et al. discovered that heat stress elevated the expression of METTL14 in abdominal AT in piglets [51]. Furthermore, the METTL14 expression levels in the dorsal fat of piglets were substantially reduced by providing a diet rich in branched-chain amino acids [52]. The current investigation is the first to elucidate the levels of METTL14 in ATs of obese and lean animals. We found that, at almost all tested weeks of age, METTL14 expression was markedly amplified in abdominal AT in fat chickens compared to lean chickens (Figure 1A–C). Moreover, it was found that METTL14 mRNA and protein levels showed a decreasing trend during preAC differentiation (Figure 1H–J), which was inconsistent with previous research in mice [26]. The findings suggest that there may be differences between birds and mammals in the regulation of METTL14 expression during preAC differentiation.

PreAC differentiation is a multifaceted process controlled by a network of TFs. PPARγ is the most crucial TF regulating mammals’ adipogenesis [53]. Earlier studies have also demonstrated that PPARγ is a key factor in preAC differentiation in birds [54]. C/EBPα is widely recognized as the main driver of preAC differentiation, and it functions in combination with PPARγ to determine the gene expression profile of mature adipocytes [55]. A-FABP is exclusively expressed in adipocytes and is vital for fatty acid transport and preAC differentiation [56]. A-FABP is regarded as a late marker of adipogenesis due to its increasing expression level throughout adipocyte differentiation [57]. In this study, we used loss-of-function and gain-of-function approaches to investigate the function of METTL14 in the differentiation of preACs in chickens. The findings demonstrated that METTL14 knockdown resulted in reduced lipid droplets and *chPPARγ*, *chC/EBPα*, and *chA-FABP* levels (Figure 2 and Figure 3), while METTL14 overexpression reversed these effects (Figure 4). These results were consistent with those of Kobayashi et al. and Liu et al., who showed that knockdown of *METTL14* impedes lipid droplet accumulation in preACs in mice and bovines [26,27]. The current findings revealed that the function of METTL14 in preAC differentiation is conserved between birds and mammals. In addition, Kobayashi et al. also found that METTL14 promoted cell cycle transition in MCE during mouse 3T3-L1 preAC differentiation [26]. It should be noted that the process of mouse preAC differentiation in vitro includes four steps: proliferation, growth arrest, MCE, and terminal differentiation [58]. However, whether growth arrest and MCE are involved in the chicken preAC differentiation in vitro remains unknown. Therefore, we were unable to investigate the METTL14 role in MCE during preAC differentiation in chicken. In addition, although this study reveals the function of METTL14 in chicken preadipocyte differentiation using the in vitro cell model, with the purpose of further investigating the effect of METTL14 on chicken abdominal fat deposition, in vivo experiments should be conducted in the future, such as generating *chMETTL14* gene-edited chickens.

Here, 60 potential gene targets of METTL14 were found by means of MeRIP-seq and RNA-seq integrated analysis. We mainly focused on specific genes whose mRNA levels were altered due to the m^6^A modification change. However, it is crucial to consider that genes without a change in mRNA levels may also be the targets of METTL14-dependent m^6^A modification in preAC differentiation, because m^6^A modification can not only affect mRNA levels by regulating mRNA stability; they also influence protein levels by modulating translation efficiency [15]. In addition, our MeRIP-seq data demonstrated that METTL14 overexpression had no impact on the mRNA m^6^A levels of *chPPARγ*, *chC/EBPα*, and *chA-FABP* (Table S2). We considered that METTL14 overexpression altered the expression of *chPPARγ*, *chC/EBPα*, and *chA-FABP* (Figure 4G) for two possible reasons: (1) METTL14 regulates the expression of *chPPARγ*, *chC/EBPα*, and *chA-FABP* indirectly. (2) It has been reported that METTL14 can regulate gene transcription by binding to genomic functional elements [59,60]. Therefore, we speculated that METTL14 may promote the expression of *chPPARγ*, *chC/EBPα*, and *chA-FABP* by binding to and activating their enhancers or/and promoters. It is necessary to extensively explore the underlying mechanism by which METTL14 regulates the expression of *chPPARγ*, *chC/EBPα*, and *chA-FABP* in the future. Next, we found that *chBMI1*, *chTMED3*, and *chMFAP2* were the critical targets of METTL14 by RT-qPCR, MeRIP-qPCR, and gene expression-related analysis (Figure 5E–J). Our mutagenesis and luciferase reporter assays indicated that the m^6^A sites located in the 3′ UTRs of *chBMI1* (A2773), *chTMED3* (A1221), and *chMFAP2* (A850) were crucial for METTL14-mediated post-transcriptional regulation of their expression (Figure 6). It should be noted that, due to the long 3′ UTR sequences of *chBMI1*, *chTMED3*, and *chMFAP2*, we only inserted part of the 3′ UTR to the vector in mutagenesis and luciferase reporter assays. Therefore, it cannot be ruled out that METTL14 may also exert its regulatory effect on *chBMI1*, *chTMED3*, and *chMFAP2* through other m^6^A sites. Furthermore, the effects observed in the 3′ UTR mutation analysis might not be entirely attributable to m^6^A modification, because an A-to-T mutation might also alter RNA secondary structure, RNA binding and recognition sites, and miRNA binding sites.

PRC1 mainly promotes the formation of facultative heterochromatin by compressing chromatin structure and catalyzing the formation of H2AK119ub1 modification, thereby maintaining the silencing state of PRC1 target genes [61]. BMI1 is an important part of the PRC1 complex, and it is involved in cell apoptosis, DNA repair, cardiac progenitor differentiation, and tumor development [62,63,64]. To date, no evidence has been reported on whether BMI1 is involved in regulating preAC differentiation. In this study, we found that silencing *chBMI1* reduced lipid droplet accumulation and *chA-FABP* and *chPPARγ* expression in chicken preACs (Figure 8A,B), which was inconsistent with previous research in mice that BMI1 knockout could promote the expression of *PPARγ* and lipid droplet accumulation in mouse BMSCs [43,65]. The current results suggested that BMI1 might have distinct functions at different adipogenic stages. TMED3 is located in membranes where it is involved in bidirectional transport between the Golgi apparatus and the endoplasmic reticulum [66]. Currently, there is limited research on TMED3, primarily focused on its relevance to cancer. TMED3 can improve the activity of the Wnt/β-catenin axis in both breast and lung cancer [47]. So far, it is unknown whether TMED3 is involved in regulating adipogenesis. Herein, we found that silencing of *chTMED3* increases lipid droplets and *chA*-*FABP* and *chPPARγ* expression in chicken preACs (Figure 8C,D). MFAP2 is an extracellular matrix protein of connective-tissue microfibrils that plays a role in microfibril assembly, elastin formation, and tissue homeostasis [67]. MFAP2 is involved in bone formation, vascular development, and liver fibrosis [68,69,70]. It has been found that MFAP2 promotes liver fibrosis in hepatic stellate cells by activating the SMAD3 axis mediated by TGFβ [70]. Knockout of *MFAP2* in mice is associated with fat cell hypertrophy and susceptibility to diabetes [46,71]. These findings show that MFAP2 is involved in fat deposition, but there is no report on whether MFAP2 regulates preAC differentiation. Here, MFAP2 silencing enhanced lipid droplet accumulation and *chA-FABP* and *chPPARγ* mRNA levels in chicken preACs (Figure 8E,F), a finding which is analogous to the earlier research results [46,71] on *MFAP2* gene knockout mice. Notably, we also demonstrated that *chBMI1*, *chTMED3*, and *chMFAP2* were functionally essential targets of METTL14 in adipogenesis (Figure 9). Taken together, we have revealed, for the first time, that BMI1, TMED3, and MFAP2 are novel regulators of preAC differentiation and underscored their important roles in METTL14-mediated adipogenesis. However, it is still unclear how BMI1, TMED3, and MFAP2 regulate chicken preadipocyte differentiation. For instance, can BMI1 regulate ubiquitination of H2AK119 and chromatin accessibility in the promoter region of adipogenesis-related genes? Can TMED3 and MFAP2 activate the Wnt/β-catenin and TGFβ-SMAD3 axes, respectively, in chicken adipogenesis? Further in-depth research is needed to address these issues.

## 5. Conclusions

In summary, we demonstrated that METTL14 promotes chicken preAC differentiation through a dual-reader m^6^A mechanism that stabilizes *chBMI1* mRNA via IGF2BP1/2 while degrading *chTMED3* and *chMFAP2* mRNAs via YTHDF2. Our findings not only reveal the inter-species conservation of METTL14 function during preadipocyte differentiation; they also establish a mechanistic framework for the understanding of how an m^6^A methyltransferase achieves target-specific regulation through reader diversification in adipogenesis.

## Figures and Tables

**Figure 1:**
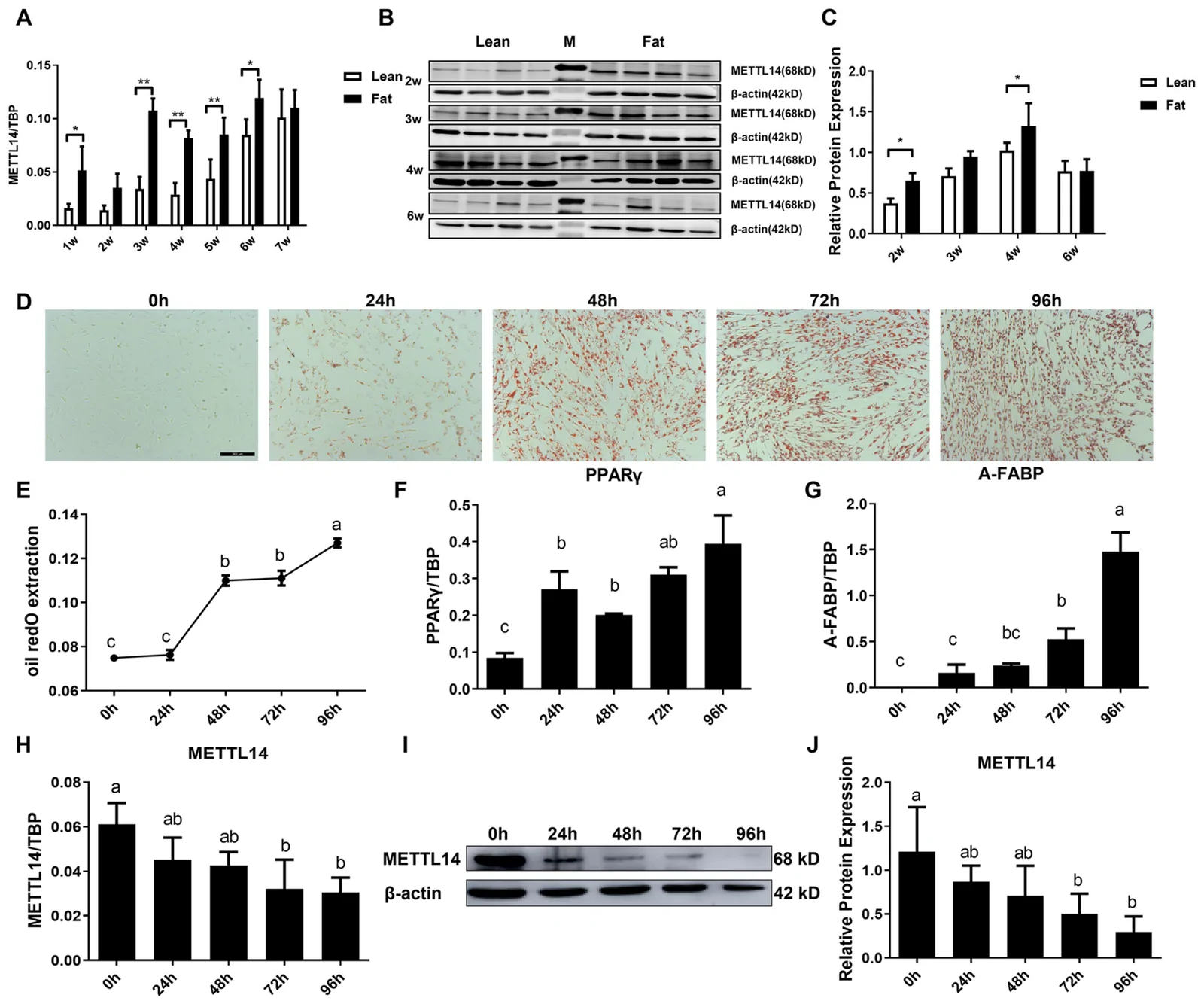
METTL14 expression pattern in adipose tissue and during preAC differentiation. mRNA (**A**) and protein (**B**) expression of *chMETTL14* in the abdominal adipose tissue of lean and fat G_19_ broilers, assessed by RT-qPCR and WB (*n* = 4). M, protein marker. (**C**) WB data quantification (*n* = 4). (**D**) ORO staining over 0 to 96 h of ICP2 cells’ differentiation. Scale bar: 200 μm. (**E**) ORO extraction assay (*n* = 3). (**F**–**H**) mRNA levels of *chPPARγ*, *chA-FABP*, and *chMETTL14* during ICP2 cells differentiation measured by RT-qPCR (*n* = 3). (**I**) METTL14 protein levels during ICP2 cell differentiation determined by WB. (**J**) WB data quantification (*n* = 3). Data are mean ± SD. ^a–c^ Different lowercase letters indicate significant differences across time points (*p* < 0.05). ** *p* < 0.01; * *p* < 0.05.

**Figure 2:**
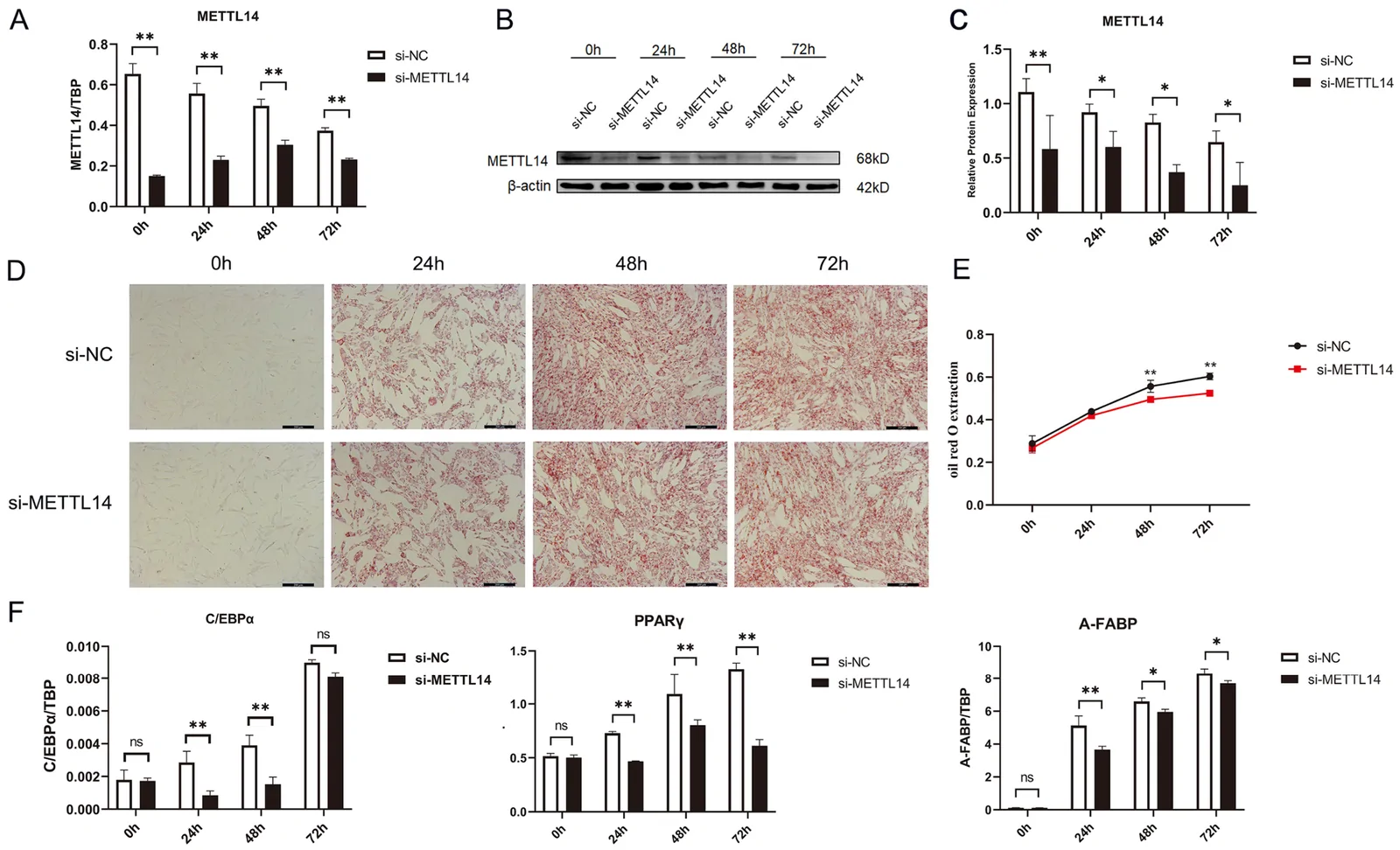
Effects of knockdown of *chMETTL14* on the differentiation of ICP2 cells. RT-qPCR (**A**) and WB (**B**) assays for *chMETTL14* mRNA and protein expression in ICP2 cells with or without *chMETTL14* knockdown. (**C**) WB data quantification. Accumulation of lipid droplets measured by ORO staining (**D**) and extraction assay (**E**). Scale bar: 200 μm. (**F**) *chC/EBPα*, *chPPARγ*, and *chA-FABP* mRNA levels measured via RT-qPCR. *n* = 3. Data are presented as mean ± SD. * *p* < 0.05; ** *p* < 0.01; ns, not significant.

**Figure 3:**
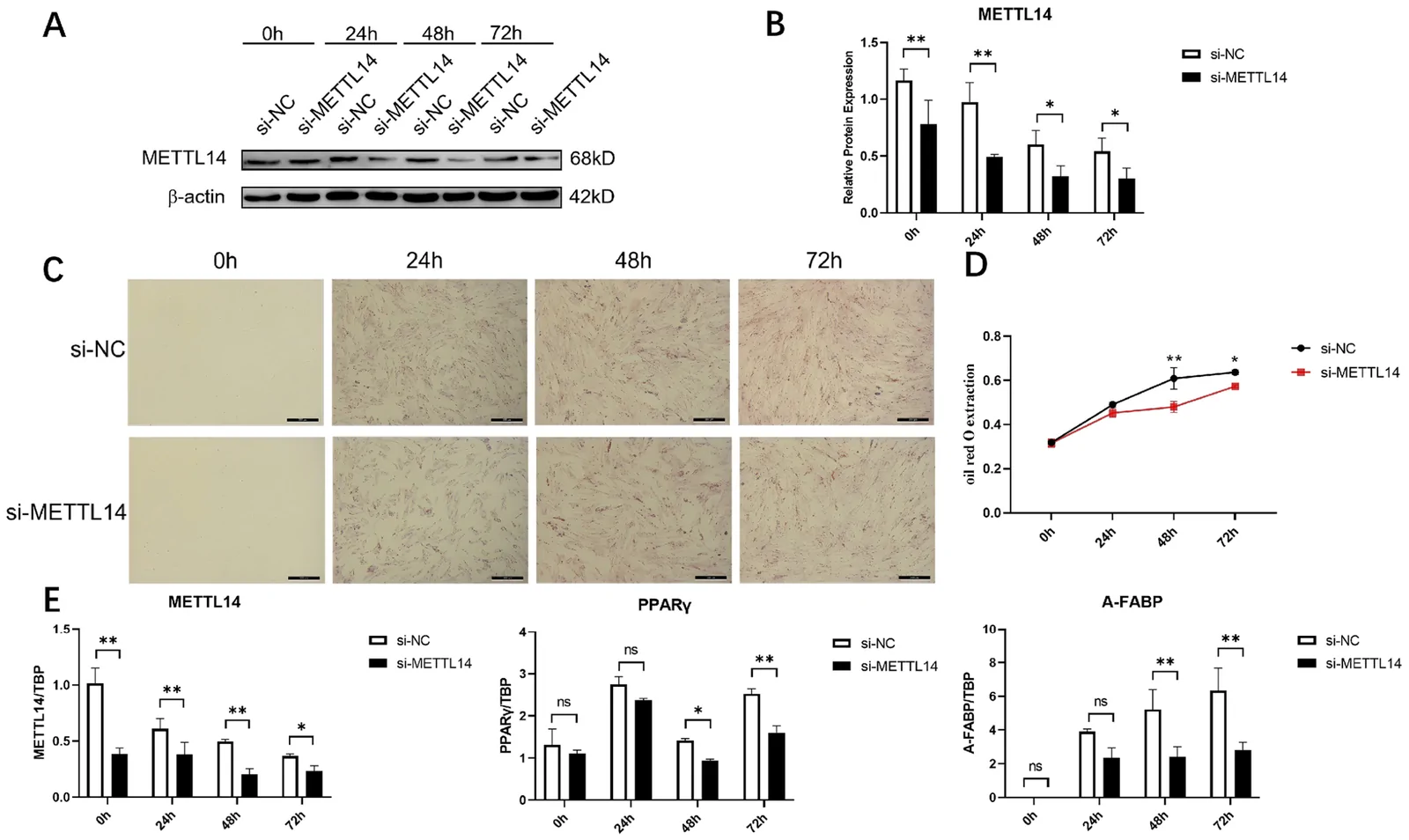
Effects of knockdown of *chMETTL14* on the differentiation of chicken primary preadipocytes. (**A**) WB assays for METTL14 protein expression in chicken primary preadipocytes with or without *chMETTL14* knockdown. (**B**) WB data quantification. Accumulation of lipid droplets measured by ORO staining (**C**) and extraction assay (**D**). Scale bar: 200 μm. (**E**) *chMETTL14*, *chPPARγ*, and *chA-FABP* mRNA levels measured via RT-qPCR. *n* = 3. Data are presented as mean ± SD. * *p* < 0.05; ** *p* < 0.01; ns, not significant.

**Figure 4:**
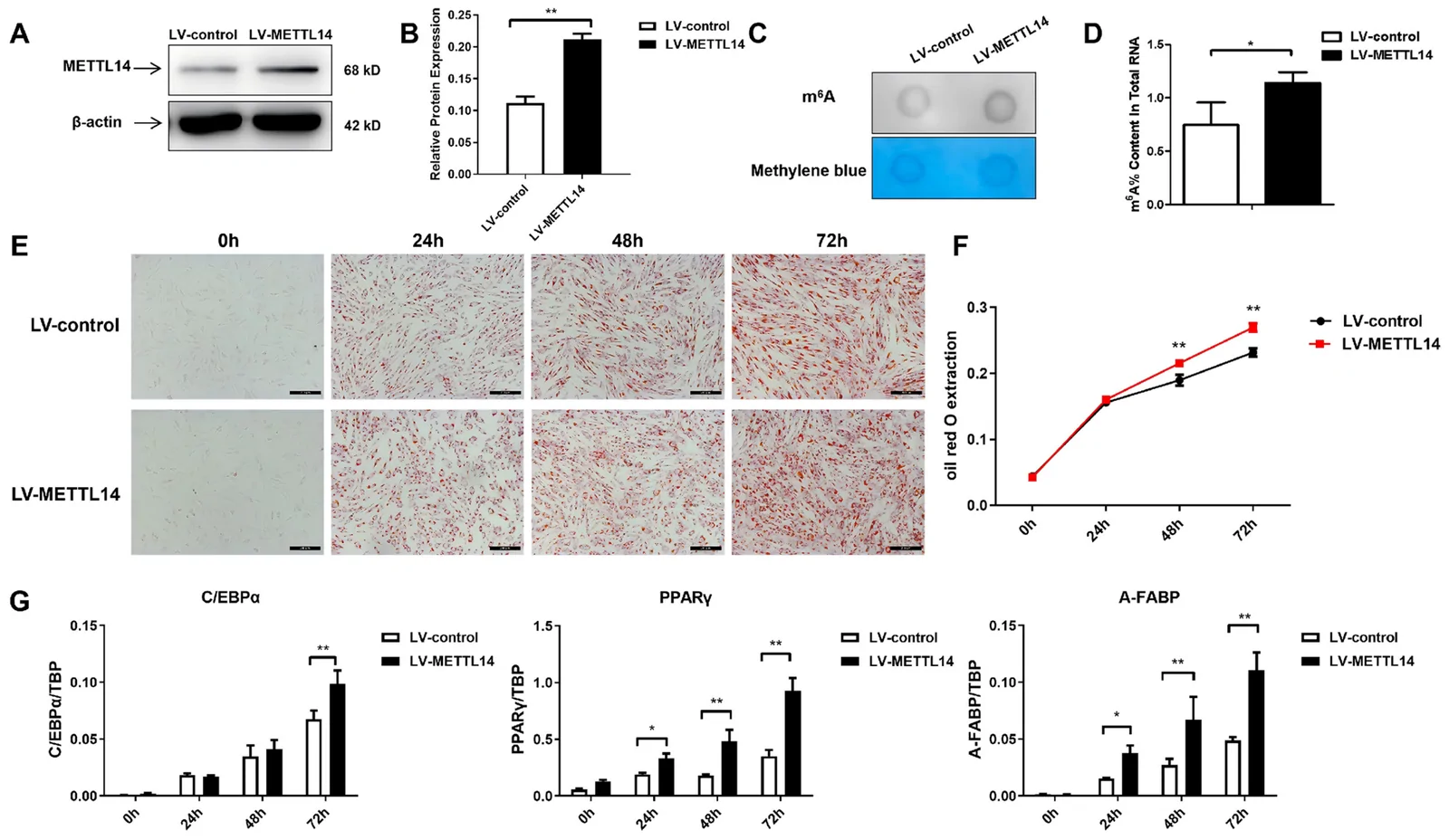
Impact of forced METTL14 expression on the chicken preAC differentiation. (**A**) WB assay of METTL14 expression in chicken ICP2 cells without or with forced expression of METTL14. (**B**) WB data quantification. The m^6^A dot blot assay (**C**) and relative quantitative information (**D**) of global m^6^A abundance in total RNA of the cell line with or without forced expression of METTL14. Accumulation of lipid droplets measured by ORO staining (**E**) and extraction assay (**F**). Scale bar: 200 μm. (**G**) *chC/EBPα*, *chPPARγ*, and *chA-FABP* mRNA levels measured by RT-qPCR. *n* = 3. Data are presented as mean ± SD. ** *p* < 0.01; * *p* < 0.05.

**Figure 5:**
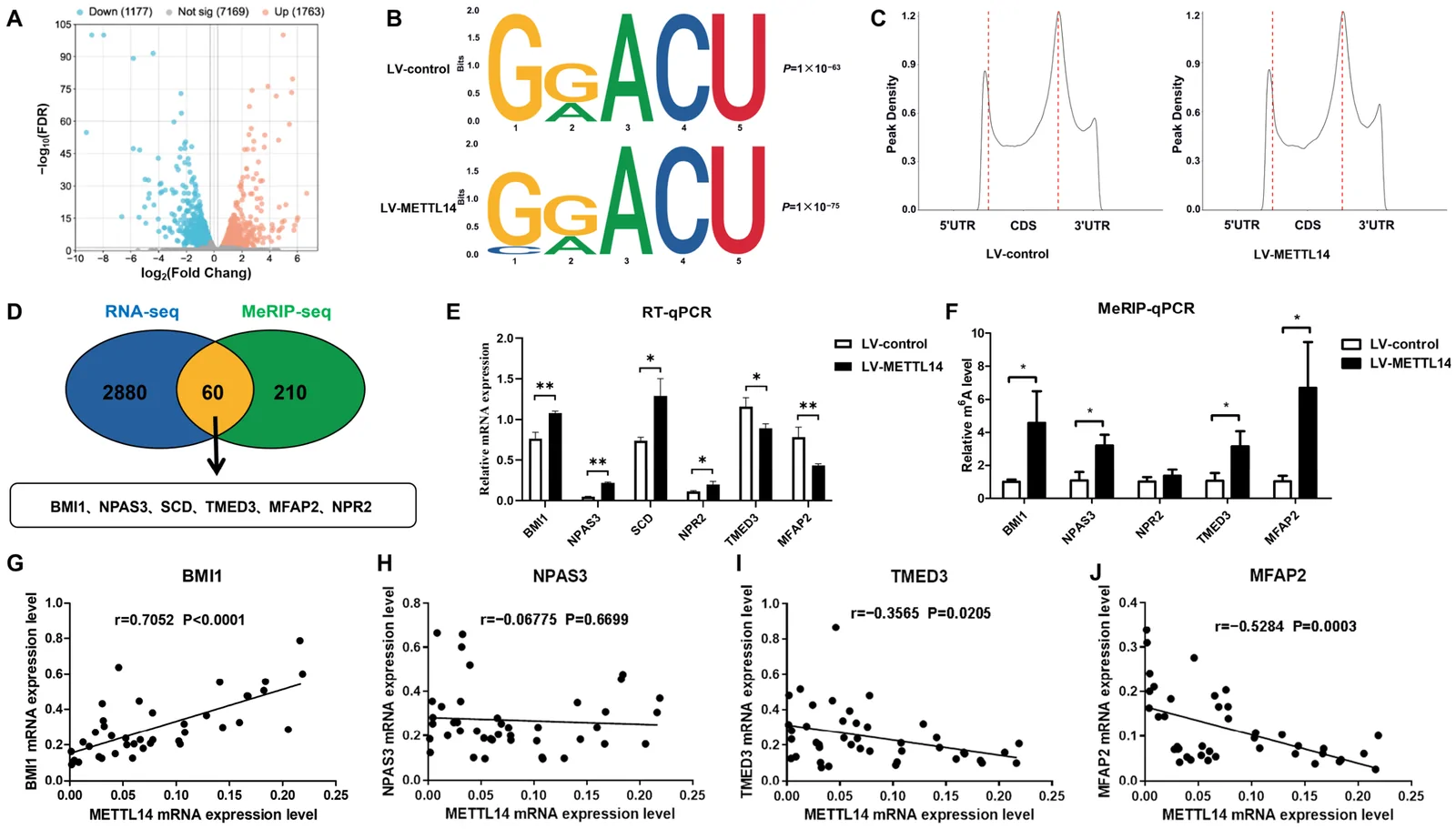
Identification of potential targets of METTL14 in the differentiated preadipocytes via RNA-seq and MeRIP-seq. (**A**) DEGs in LV-METTL14 cells compared with the LV-control cells. (**B**) Top consensus motifs identified from MeRIP-seq peaks in LV-METTL14 and LV-control cells. (**C**) Enrichment of m^6^A signals along mRNA transcripts in LV-METTL14 and LV-control cells. (**D**) MeRIP-seq and RNA-seq integrative analysis identified downstream targets of METTL14. (**E**) The mRNA levels of *chBMI1*, *chNPAS3*, *chSCD*, *chNPR2*, *chTMED3*, and *chMFAP2* in the LV-METTL14 and LV-control cells were assessed by RT-qPCR (*n* = 3). (**F**) MeRIP-qPCR analysis of m^6^A level in mRNA transcripts of *chBMI1*, *chNPAS3*, *chNPR2*, *chTMED3*, and *chMFAP2* in LV-METTL14 and LV-control cells (*n* = 3). (**G**) Correlation between *chMETTL14* and *chBMI1* expression (*N* = 42). (**H**) Correlation between *chMETTL14* and *chNPAS3* expression (*N* = 42). (**I**) Correlation between *chMETTL14* and *chTMED3* expression (*N* = 42). (**J**) Correlation between *chMETTL14* and *chMFAP2* expression (*N* = 42). Data are presented as mean ± SD. ** *p* < 0.01; * *p* < 0.05.

**Figure 6:**
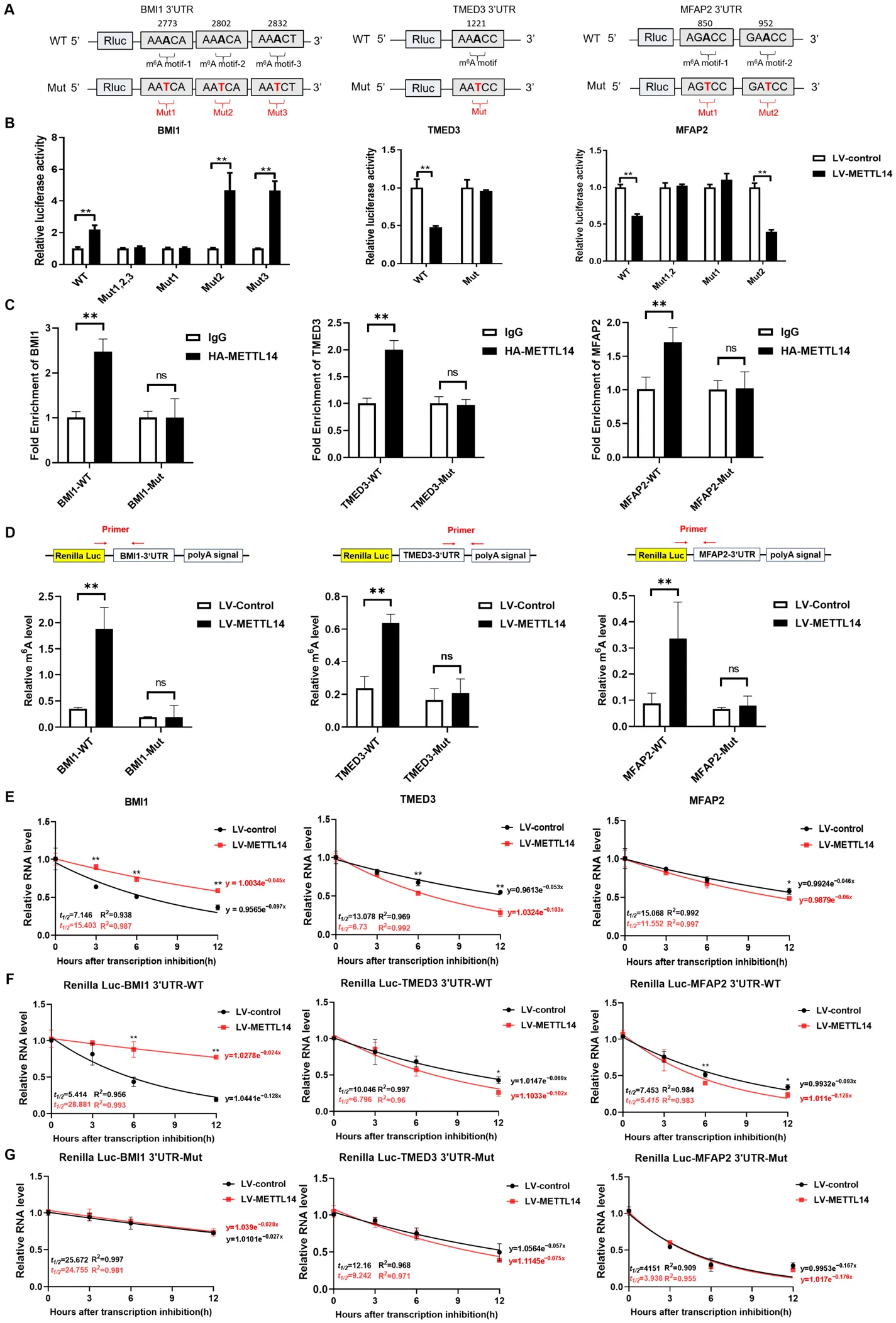
METTL14 overexpression enhances *chBMI1* mRNA stability and reduces *chTMED3* and *chMFAP2* mRNA stability via an m^6^A-dependent mechanism. (**A**) psi-Check2-Rluc-BMI1-3′UTR (left panel), psi-Check2-Rluc-TMED3-3′UTR (middle panel), and psi-Check2-Rluc-MFAP2-3′UTR (right panel) with either WT or mutant (A-to-T mutation) m^6^A sites. The number above the box represents the position of the m^6^A motif in the mRNA of *chBMI1*, *chTMED3*, or *chMFAP2*. (**B**) LV-METTL14 or LV-control cells were transfected with WT or mutated plasmids of psi-Check2-Rluc-BMI1-3′UTR, psi-Check2-Rluc-TMED3-3′UTR, and psi-Check2-Rluc-MFAP2-3′UTR. *Renilla* luciferase activity was normalized to that of Firefly. (**C**) The physical binding of METTL14 to the *Renilla* luciferase gene with WT and mutant 3′ UTR of *chBMI1*, *chTMED3*, or *chMFAP2* was analyzed using the RIP assay in LV-METTL14 cells. (**D**) Luciferase reporter assay-related gene-specific m^6^A-qPCR analysis of m^6^A levels in exogenous mRNA transcripts of *Renilla* Luc-BMI1 3′UTR, *Renilla* Luc-TMED3 3′UTR, or *Renilla* Luc-MFAP2 3′UTR in LV-METTL14 and LV-control cells. (**E**) RT-qPCR determination of *chBMI1*, *chTMED3*, and *chMFAP2* levels in LV-METTL14 and LV-control cells after actinomycin D treatment. (**F**,**G**) Luciferase reporter assay-related RT-qPCR analysis of expression levels in exogenous mRNA transcripts of *Renilla* Luc-BMI1 3′UTR, *Renilla* Luc-TMED3 3′UTR, or *Renilla* Luc-MFAP2 3′UTR in LV-METTL14 and LV-control cells after actinomycin D treatment. *n* = 3. Data are presented as mean ± SD. ** *p* < 0.01; * *p* < 0.05; ns, not significant.

**Figure 7:**
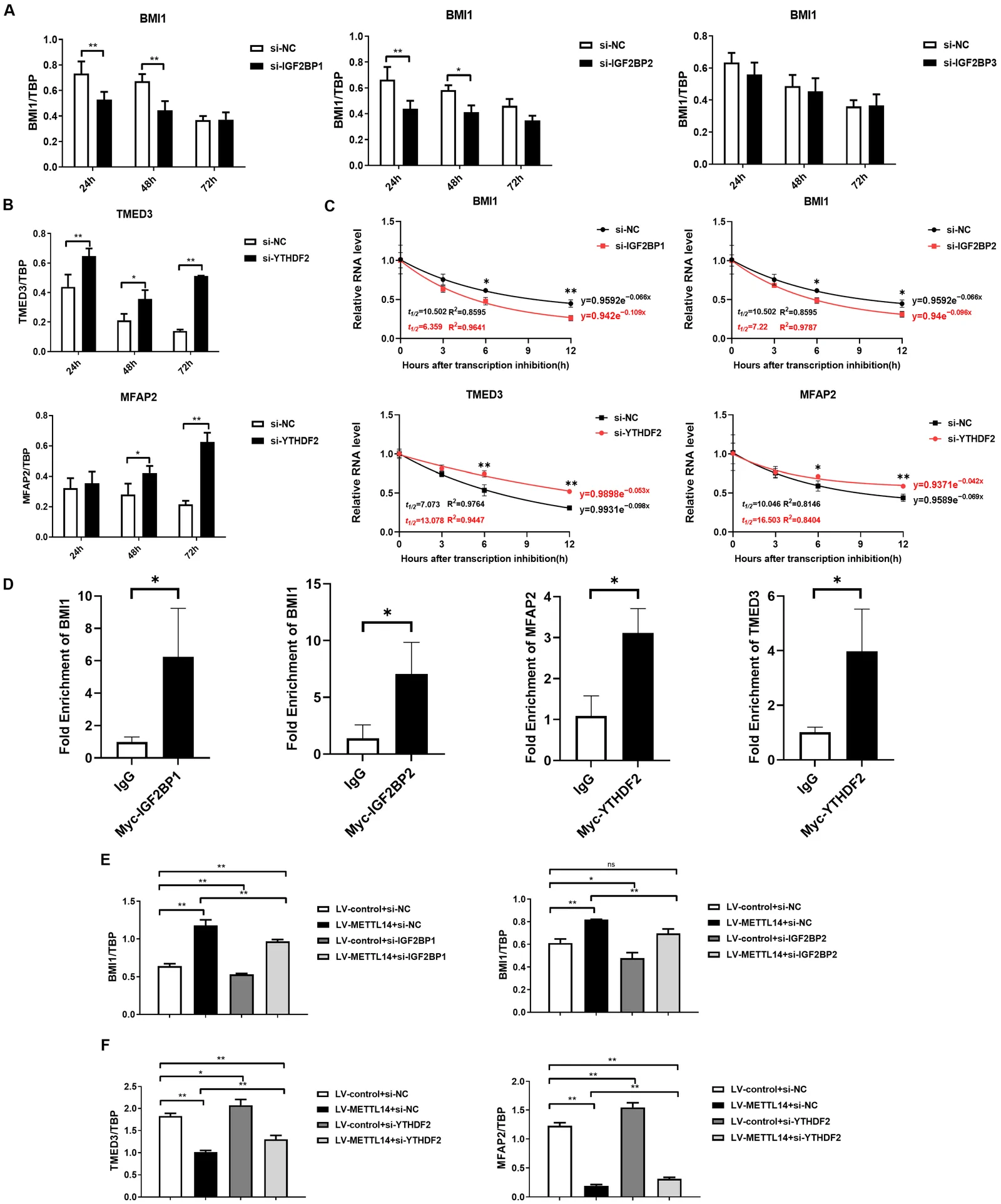
m^6^A readers participate in the regulation of the expression of BMI1, TMED3, and MFAP2 by METTL14. (**A**) RT-qPCR determination of *BMI1* levels after m^6^A readers, IGF2BP1, IGF2BP2, and IGF2BP3 depletion in ICP2 cells. (**B**) RT-qPCR determination of *chTMED3* and *chMFAP2* expression after m^6^A reader YTHDF2 depletion in ICP2 cells. (**C**) RT-qPCR determination of *chBMI1*, *chTMED3*, and *chMFAP2* levels in ICP2 cells with actinomycin D treatment after m^6^A readers, IGF2BP1, IGF2BP2, and YTHDF2 depletion. (**D**) The physical binding of IGF2BP1/2 to the *chBMI1* transcript and the binding of YTHDF2 to *chTMED3* and *chMFAP2* transcripts were analyzed using the RIP assay in LV-METTL14 cells at 48 h after differentiation. (**E**) RT-qPCR determination of *chBMI1* levels in LV-METTL14 and LV-control cells in the presence or absence of IGF2BP1/2 knockdown 48 h after differentiation. (**F**) RT-qPCR determination of *chTMED3* and *chMFAP2* levels in LV-METTL14 and LV-control cells with or without YTHDF2 knockdown 48 h after differentiation. *n* = 3. Data are presented as mean ± SD. ** *p* < 0.01; * *p* < 0.05; ns, not significant.

**Figure 8:**
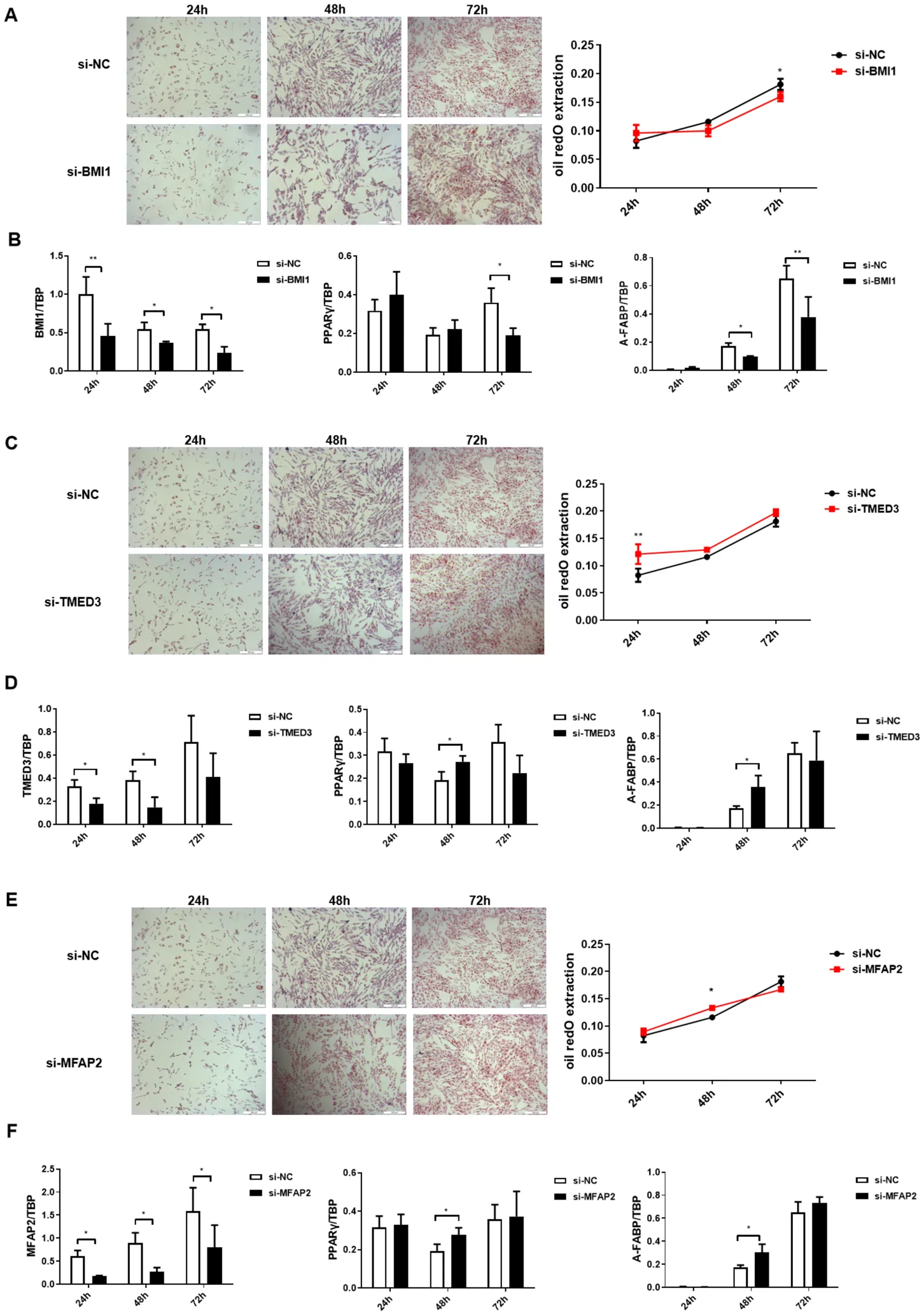
The effects of knockdown of *chBMI1*, *chTMED3*, or *chMFAP2* on the preAC differentiation of chicken. (**A**,**C**,**E**) Accumulation of lipid droplets measured by ORO staining and extraction assay. Scale bar: 200 μm. (**B**,**D**,**F**) *chPPARγ* and *chA-FABP* mRNA levels measured via RT-qPCR. *n* = 3. Data are presented as mean ± SD. ** *p* < 0.01; * *p* < 0.05.

**Figure 9:**
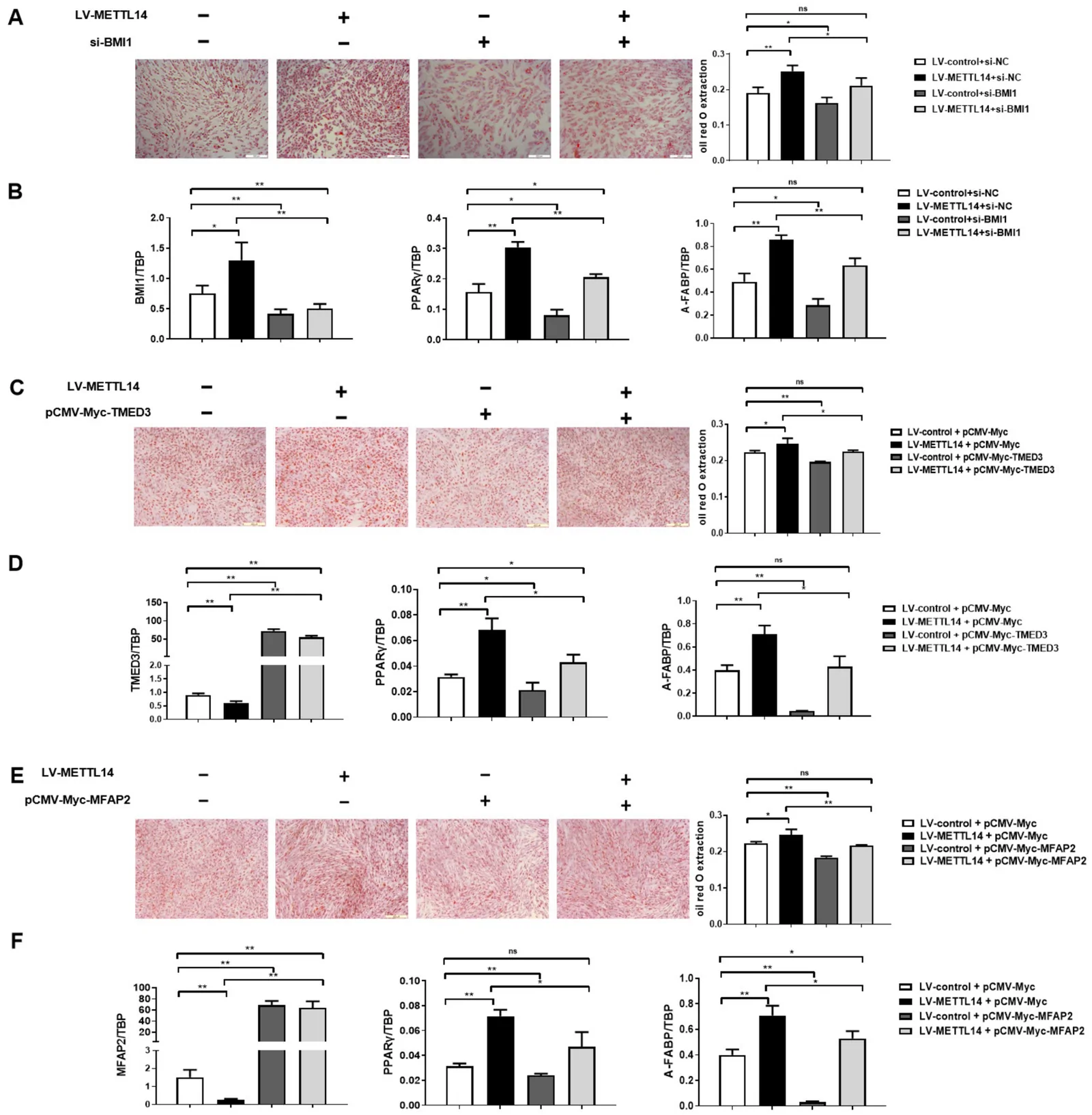
*chBMI1*, *chTMED3*, and *chMFAP2* are key METTL14 targets in chicken preAC differentiation. (**A**,**C**,**E**) Accumulation of lipid droplets measured by ORO staining and extraction assay. Scale bar: 200 μm. (**B**,**D**,**F**) *chPPARγ* and *chA-FABP* mRNA levels measured via RT-qPCR. *n* = 3. Data are presented as mean ± SD. ** *p* < 0.01; * *p* < 0.05; ns, not significant.

## Data Availability

The datasets presented in this study can be found in an online repository. The name of the repository and accession number can be found below: [NCBI SRA and PRJNA1173397 (https://dataview.ncbi.nlm.nih.gov/object/PRJNA1173397?reviewer=ojous0ia7en0biuf1neec4a1d2; accessed 26 September 2026)].

## Supplementary Materials

The following supporting information can be downloaded at: https://www.mdpi.com/article/10.3390/cells15191761/s1, Supplementary Material 1—Main: This is the main file that contains the Supplementary Figures and Tables. Figure S1: The AFP of G_19_ and G_24_ birds used in this study. Figure S2: Screening of effective and specific interference fragments of *chMETTL14*, *chIGF2BP1*, *chIGF2BP2*, *chIGF2BP3*, *chYTHDF2*, *chBMI1*, *chTMED3*, and *chMFAP2*. Figure S3: The effect of METTL14 overexpression on expression levels and m^6^A levels of *chBMI1*, *chTMED3*, and *chMFAP2* in chicken primary preadipocytes 72 h after differentiation. Figure S4: The m^6^A abundances in *chBMI1*, *chTMED3*, and *chMFAP2* mRNA transcripts in METTL14-overexpressing (METTL14_1, METTL14_2, and METTL14_3) and control (Control_1, Control_2, and Control_3) ICP2 cells, as shown by MeRIP-seq. Figure S5: Verification of expression of Myc-TMED3 and Myc-MFAP2 in the extracts from ICP2 cells at 48 h after transfection. Table S1: The sequence of siRNAs used in this study. Table S2: RT-qPCR primer sequences. Table S3: MeRIP-qPCR primer sequences. Table S4: Primer sequences of luciferase reporter-related gene-specific m^6^A-qPCR. Table S5: Primer sequences for RIP-qPCR. Table S6: The m^6^A peak coordinates for BMI1, TMED3, and MFAP2. Supplementary Material 2—Main: This is the main file that contains the Supplementary Data. Data S1: Differentially expressed genes, identified by RNA-seq, between LV-METTL14 and LV-control cells. Data S2: m^6^A peaks in the LV-control cells. Data S3: m^6^A peaks in the LV-METTL14 cells. Data S4: Differential m^6^A methylation genes, identified by MeRIP-Seq, between LV-METTL14 and LV-control cells.
