# Supplementary material for: METTL14 Promotes Chicken Preadipocyte Differentiation by Modulating mRNA Stability via a Dual-Reader m6A Mechanism
Source: Cells. 2026 Sep 27;15(19):1761. doi: 10.3390/cells15191761 (PMC13664386; doi:10.3390/cells15191761)
Supplement: Supplementary file 1 [file cells-15-01761-s001.zip › Supplementary Material 1-Main.pdf]

Supplementary Material 1-Main

METTL14 promotes chicken preadipocyte differentiation by modulating mRNA stability via a dual-reader m^{6}A mechanism

Saibo Zhang [1,2,3,†], Qi Zhang [1,2,3,4,5,†], Yawen Yang [1,2,3], Chang Liu [1,2,3], Fei Teng [1,2,3], Shouzhi Wang [1,2,3], Hui Li [1,2,3,*] and Bohan Cheng [1,2,3,*]

[1] *Key Laboratory of Chicken Genetics and Breeding, Ministry of Agriculture and Rural Affairs, Harbin 150030, Heilongjiang, China*

[2] *Key Laboratory of Animal Genetics, Breeding and Reproduction, Education Department of Heilongjiang Province, Harbin 150030, Heilongjiang, China*

[3] *College of Animal Science and Technology, Northeast Agricultural University, Harbin 150030, Heilongjiang, China*

[4] *Key Laboratory of Exploration and Innovative Utilization of White Goose Germplasm Resources in the Cold Region of Heilongjiang Province, #2 Xinyang Road, New Development District, Daqing 163319, Heilongjiang, China*

[5] *College of Animal Science and Technology, Heilongjiang Bayi Agricultural University, #2 Xinyang Road, New Development District, Daqing 163319, Heilongjiang, China*

***Corresponding authors.**

Hui Li & Bohan Cheng

E-mail addresses: lihui@neau.edu.cn (H. Li), bhcheng@neau.edu.cn (B. Cheng).

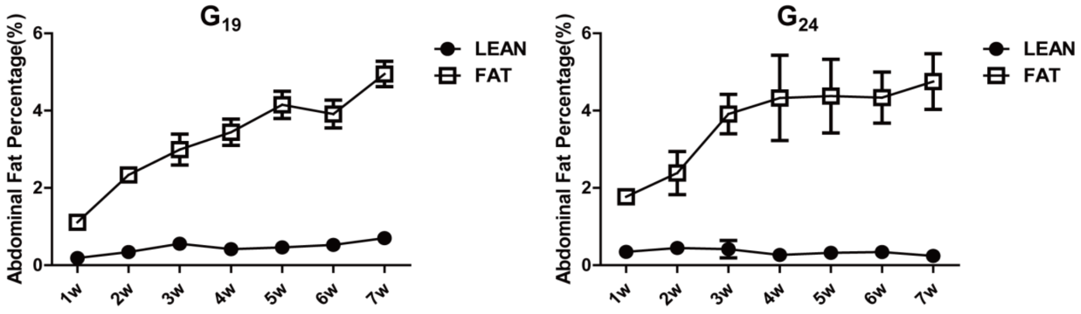

Figure S1. The AFP of G_{19} and G_{24} birds used in the study.

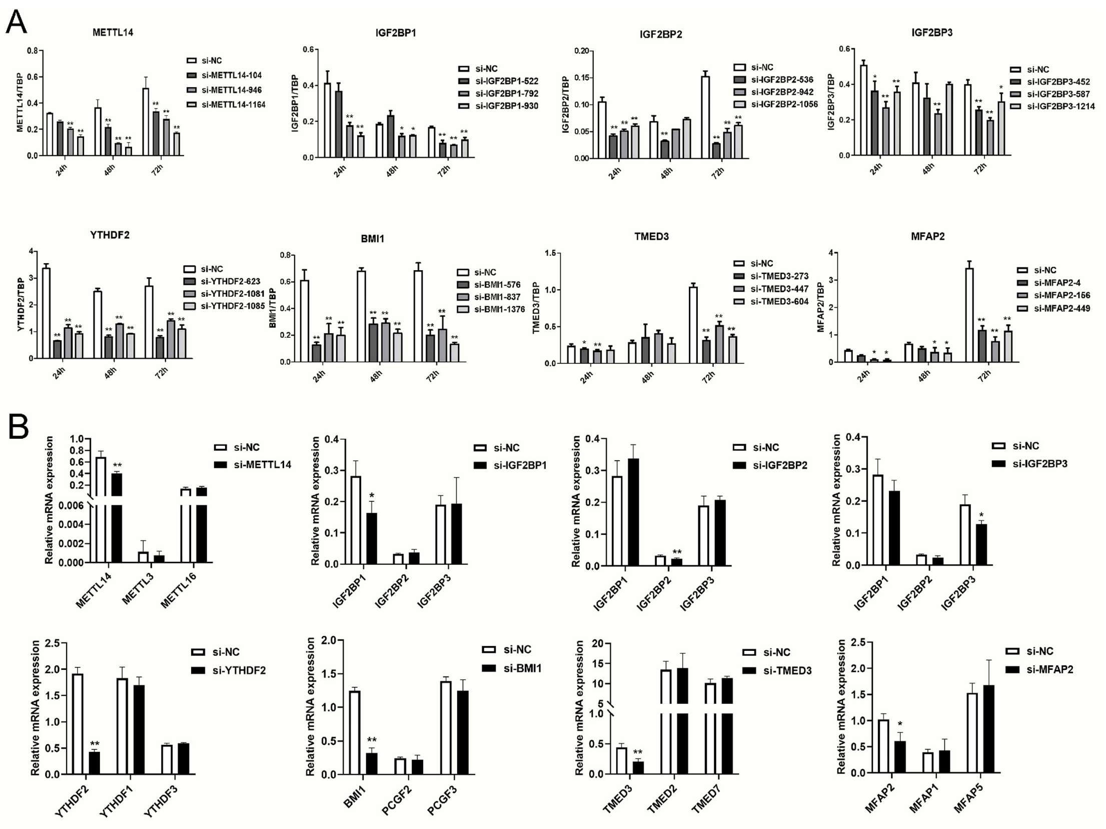

Figure S2. Screening of effective and specific interference fragments of *chMETTL14*, *chIGF2BP1*, *chIGF2BP2*, *chIGF2BP3*, *chYTHDF2*, *chBMI1*, *chTMED3* and *chMFAP2*. (A) Screening of effective interference fragments of *chMETTL14*, *chIGF2BP1*, *chIGF2BP2*, *chIGF2BP3*, *chYTHDF2*, *chBMI1*, *chTMED3* and *chMFAP2* at 24, 48, and 72 h after transfection. (B) Detection of the specificity of interference fragment of *chMETTL14*, *chIGF2BP1*, *chIGF2BP2*, *chIGF2BP3*, *chYTHDF2*, *chBMI1*, *chTMED3* and *chMFAP2* at 48 h after transfection. n=3. Data are mean ± SD. $^{*}P < 0.05$, $^{**}P < 0.01$.

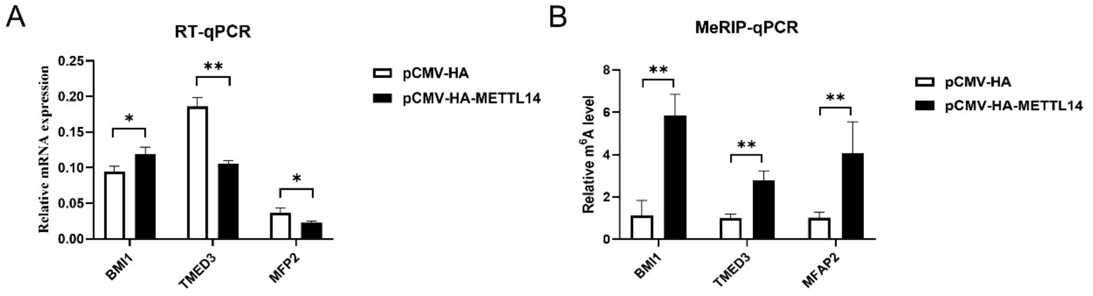

Figure S3. The effect of METTL14 overexpression on expression levels and m^{6}A levels of *chBMI1*, *chTMED3* and *chMFAP2* in chicken primary preadipocytes at 72 h after differentiation. (A) The mRNA levels of *chBMI1*, *chTMED3* and *chMFAP2* in chicken primary preadipocytes transfected with pCMV-HA-METTL14 or pCMV-HA empty vector were assessed by RT-qPCR. (B) MeRIP-qPCR analysis of m^{6}A level in mRNA transcripts of *chBMI1*, *chTMED3* and *chMFAP2* in chicken primary preadipocytes transfected with pCMV-HA-METTL14 or pCMV-HA empty vector. n=3. Data are mean ± SD. $^*P < 0.05$, $^{**}P < 0.01$.

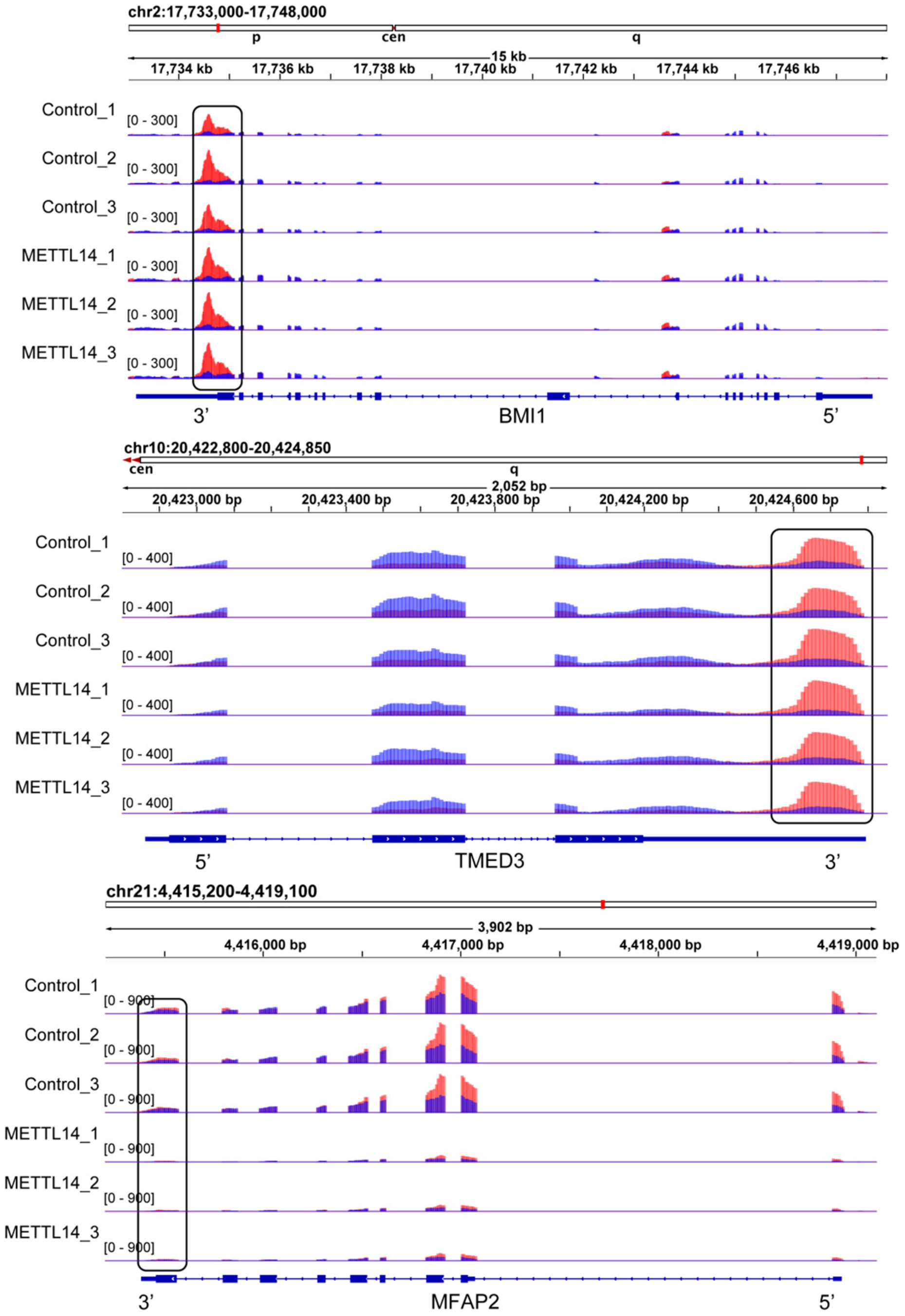

Figure S4. The m^{6}A abundances in *chBMI1*, *chTMED3* and *chMFAP2* mRNA transcripts in METTL14-overexpressing (METTL14_1, METTL14_2 and METTL14_3) and control (Control_1, Control_2 and Control_3) ICP2 cells as shown by MeRIP-seq. The m^{6}A peaks shown in the black rectangles are those that have a markedly higher abundance in METTL14_1/2/3 than in Control_1/2/3 cells.

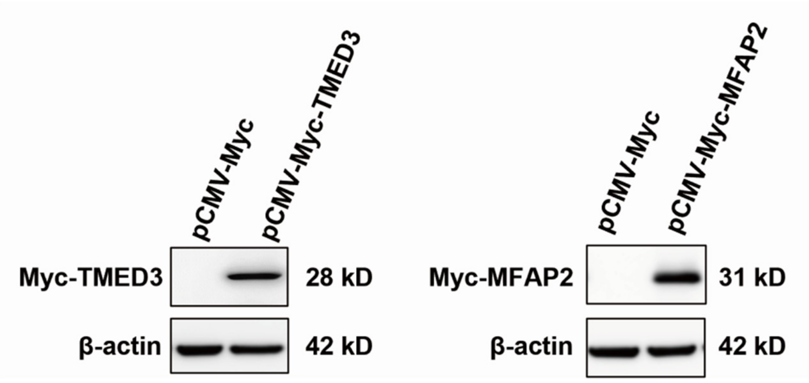

Figure S5. Verification of expression of Myc-TMED3 and Myc-MFAP2 in the extracts from ICP2 cells at 48 h after transfection.

Table S1 The sequence of siRNAs used in this study

siRNA Name	Sequence（5'-3'）	
NC-siRNA	sense:	UUCUCCGAACGUGUCACGUTT
	antisense:	ACGUGACACGUUCGGAGAATT
METTL14-Gallus-104	sense:	GCUCUUGGCUCAGCAGUUATT
	antisense:	UAACUGCUGAGCCAAGAGCTT
METTL14-Gallus-946	sense:	GCAGUACAGAUGGUGACUUTT
	antisense:	AAGUCACCAUCUGUACUGCTT
METTL14-Gallus-1164	sense:	GCAGAAACGUACUCUUCAUTT
	antisense:	AUGAAGAGUACGUUUCUGCTT
MFAP2-Gallus-4	sense:	GGAUGCUCACCAAUGGCUUTT
	antisense:	AAGCCAUUGGUGAGACUCCTT
MFAP2-Gallus-156	sense:	GGGCCAAUACAGCAGGUUUTT
	antisense:	AAACCUGCUGUAUUGGCCCTT
MFAP2-Gallus-449	sense:	GCCUGAACGAGAUCUGCUUTT
	antisense:	AAGCAGAUCUCGUUCAGGCTT
TMED3-Gallus-273	sense:	GCACCAUCUACAGGGAGACTT
	antisense:	GUCUCCCUGUAGAUGGUGCTT
TMED3-Gallus-447	sense:	GCGUCACCGCUUUGACACATT
	antisense:	UGUGUCAAAGCGGUGACGCTT
TMED3-Gallus-604	sense:	GGAAACCGUCAUCCUCUUCTT
	antisense:	GAAGAGGAUGACGGUUUCCTT

BMI1-Gallus-576	sense:	GGUACUUGGAGACGAGCAATT
	antisense:	UUGCUCGUCUCCAAGUACCTT
BMI1-Gallus-837	sense:	GCUUAUCCAUUGAGUUCUUTT
	antisense:	AAGAACUCAAUGGAUAAGCTT
BMI1-Gallus-1376	sense:	GCGCGGAAAUCCUCCAUAATT
	antisense:	UUAUGGAGGAUUUCCGCGCTT
YTHDF2-Gallus-623	sense:	GCACGGCUUUAACUUCUUUTT
	antisense:	AAAGAAGUUAAAGCCGUGCTT
YTHDF2-Gallus-1085	sense:	GGAUAUAGGAACUUGGGAUTT
	antisense:	AUCCCAAGUUCCUAUAUCCTT
YTHDF2-Gallus-1801	sense:	GGCACAUCCGCCUAGAGAATT
	antisense:	UUCUCUAGGCGGAUGUGCCTT
IGF2BP1-Gallus-522	sense:	GCAAGCCAUCAUGAAGUUATT
	antisense:	UAACUUCAUGAUGGCUUGCTT
IGF2BP1-Gallus-792	sense:	GCAGACACAGUCCAAGAUUTT
	antisense:	AAUCUUGGACUGUGUCUGCTT
IGF2BP1-Gallus-930	sense:	GGACACGAAGACAGCUGAUTT
	antisense:	AUCAGCUGUCUUCGUGUCCTT
IGF2BP2-Gallus-536	sense:	GCAGUUGUCAACGUCACAUTT
	antisense:	AUGUGACGUUGACAACUGCTT
IGF2BP2-Gallus-942	sense:	GCAUGAUUCUUGAUAUCAUTT
	antisense:	AUGAUAUCAAGAAUCAUGCTT

IGF2BP2-Gallus-1056	sense:	GCCGCAACCUCAAGAAGAUTT
	antisense:	AUCUUCUUGAGGUUGCGGCTT
IGF2BP3-Gallus-452	sense:	GCUGCACGGCAAACUCAUATT
	antisense:	UAUGAGUUUGCCGUGCAGCTT
IGF2BP3-Gallus-587	sense:	GGAGAACUGUGAGCAAGUUTT
	antisense:	AACUUGCUCACAGUUCUCCTT
IGF2BP3-Gallus-1214	sense:	GCAGGACUUGACACUCUAUTT
	antisense:	AUAGAGUGUCAAGUCCUGCTT

Table S2 RT-qPCR primer sequences

Gene Name	Primer sequence (5' to 3')	product size (bp)	Amplification efficiency (%)
chMETTL14	F: AAATGCTGGACCTGGGATGAT<br>R:GGCAGTGCTCCTTGGTTCTT	241	105.36
chA-FABP	F: ATGTGCGACCAGTTTGT<br>R:TCACCATTGATGCTGATAG	143	100.80
chPPARγ	F: GTGCAATCAAAATGGAGCC<br>R:CTTACAACCTTCACATGCAT	170	102.52
chC/EBPα	F: GCGACATCTGCGAGAACG<br>R: GTACAGCGGGTCGAGCTT	266	109.62
chBMI1	F:ACTCCTTCTGCAAGACGTGC<br>R:GAGTTTTATCTGACCTGATGTTGAG	117	101.99
chNPAS3	F:GGATCCTTCCAGGCGAGAAC<br>R:AGCAGCATCTCGGGACTTTT	151	100.27
chSCD	F:GGTGATGTTCCAGCGGAGAT<br>R:GGCATTGAGCCCTAAGGTGT	151	109.83
chNPR2	F:ATGTGTTTGGGGAGAGCCTG<br>R:CTGGAAGTGCTGGTACTCGG	158	101.88
chTMED3	F:AGTGCTTCCACCAAGAGCTG<br>R:GTCTCCCTGTAGATGGTGCG	127	107.58
chMFAP2	F:AACCGGTGCAATACTCCCAG	110	108.17

	R:CTGGGACTGGAAGCGAAACT		
chYTHDF2	F:GGAAAAGGCTAAGCAGGTGTTG<br>R:GGCGTTCCTTTTTAACAGTTTCCT	122	108.33
chIGF2BP1	F:GGTCAAGTCCGGCTATGCTT<br>R:GGATTTTCCGGCTCCTTTGC	149	108.24
chIGF2BP2	F:GGCTACGCCTTCGTGGATTA<br>R:AGCTTTTTGGGCACGGAGTA	122	108.54
chIGF2BP3	F:CAGTTCCTGGTGAAGACGGG<br>R:CTATGAGTTTGCCGTGCAGC	112	109.53
Renilla Luc-BMI1-3'UTR	F:TCCGCAACTACAACGCCT<br>R:ACTTCTTAGCTCCCTCGACA	108	109.58
Renilla Luc-TMED3-3'UTR	F:TCCGCAACTACAACGCCT<br>R:ACTTCTTAGCTCCCTCGACA	108	109.58
Renilla Luc-MFAP2-3'UTR	F:TCCGCAACTACAACGCCT<br>R:ACTTCTTAGCTCCCTCGACA	108	109.58
chTBP	F:GCGTTTTGCTGCTGTTATTATGAG<br>R:TCCTTGCTGCCAGTCTGGAC	122	104.70

Table S3 MeRIP-qPCR primer sequences

**Gene Name**	**Primer sequence (5' to 3')**	**product size (bp)**	**Amplification efficiency(%)**
chBMI1	F:AGGTTCTTGCTTATTGATG<br>R:CTATAAATGAAAGTTATTCGTG	132	108.95
chNPAS3	F:ATGCCTGCAGGGAATGTGTT<br>R:TTCCGTTCCAGAGTCTGTGT	104	107.06
chNPR2	F:TGTAAATATCCCTTCCCCAG<br>R:TACAGGAGCTGGGAACAGCA	98	95.57
chTMED3	F:TCCCCTTCCCTTGGTCCC<br>R:CAGAGTCATGTGGGTTTATTGTGA	115	107.07
chMFAP2	F:ATGGCCACCAGTGGGCTCTG<br>R:ACGGTTCCTTTATAGCACCA	131	102.86

Table S4 Primer sequences of luciferase reporter-related gene specific m^{6}A-qPCR

Gene Name	Primer sequence (5' to 3')	product size (bp)	Amplification efficiency(%)
Renilla Luc-BMI1-3'UTR	F:GAACGAGCAGTAATTCTAGGCGATC<br>R:TTTTACCGTTGAAATCGAACTGTAC	134	98.47
Renilla Luc-TMED3-3'UTR	F:GCCATCTCCACCAGCACCACT<br>R:AGATATTTTATTGCGGCCAGCGG	94	109.12
Renilla Luc-MFAP2-3'UTR	F:TGAAGAACGAGCAGTAATTCTAGGCG<br>R:CTGCGTCCCGGCGAGGTTA	170	101.74

Table S5 Primer sequences for RIP-qPCR

Gene Name	Primer sequence (5' to 3')	product size (bp)	Amplification efficiency(%)
Renilla Luc-BMI1-3'UTR	F:TCCGCAACTACAACGCCT<br>R:ACTTCTTAGCTCCCTCGACA	108	109.58
Renilla Luc-TMED3-3'UTR	F:TCCGCAACTACAACGCCT<br>R:ACTTCTTAGCTCCCTCGACA	108	109.58
Renilla Luc-MFAP2-3'UTR	F:TCCGCAACTACAACGCCT<br>R:ACTTCTTAGCTCCCTCGACA	108	109.58
chBMI1	F:AGGTTCTTGCTTATTGATG<br>R:CTATAAATGAAAGTTATTCGTG	132	108.95
chTMED3	F:TCCCCTTCCCTTGGTCCC<br>R:CAGAGTCATGTGGGTTTATTGTGA	115	107.07
chMFAP2	F:ATGGCCACCAGTGGGCTCTG<br>R:ACGGTTCCTTTATAGCACCA	131	102.86

Table S6 The m^6A peak coordinates for *BMI1*, *TMED3* and *MFAP2*

Gene Name	Chromosome	Peak Start	Peak End	Peak Width (bp)
chBMI1	2	17732964	17733113	150
chTMED3	10	20424470	20424795	326
chMFAP2	21	4415210	4415991	782
